# Essential Role for FtsL in Activation of Septal Peptidoglycan Synthesis

**DOI:** 10.1128/mBio.03012-20

**Published:** 2020-12-08

**Authors:** Kyung-Tae Park, Shishen Du, Joe Lutkenhaus

**Affiliations:** a Department of Microbiology, Molecular Genetics and Immunology, University of Kansas Medical Center, Kansas City, Kansas, USA; University of Nebraska Medical Center

**Keywords:** cell division, divisome, septal ring, septation

## Abstract

Spatiotemporal regulation of septal peptidoglycan (PG) synthesis is achieved by coupling assembly and activation of the synthetic enzymes (FtsWI) to the Z ring, a cytoskeletal element that is required for division in most bacteria. In Escherichia coli, the recruitment of the FtsWI complex is dependent upon the cytoplasmic domain of FtsL, a component of the conserved FtsQLB complex. Once assembled, FtsWI is activated by the arrival of FtsN, which acts through FtsQLB and FtsA, which are also essential for their recruitment. However, the mechanism of activation of FtsWI by FtsN is not clear. Here, we identify a region of FtsL that plays a key role in the activation of FtsWI which we designate AWI (activation of FtsWI) and present evidence that FtsL acts through FtsI. Our results suggest that FtsN switches FtsQLB from a recruitment complex to an activator with FtsL interacting with FtsI to activate FtsW. Since FtsQLB and FtsWI are widely conserved in bacteria, this mechanism is likely to be also widely conserved.

## Abstract (precis)

A critical step in bacterial cytokinesis is the activation of septal peptidoglycan synthesis at the Z ring. Although FtsN is the trigger and acts through FtsQLB and FtsA to activate FtsWI the mechanism is unclear.

## INTRODUCTION

**FIG 1:**
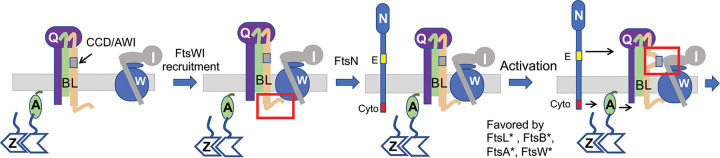
Model for recruitment and activation of FtsWI. FtsQLB localizes to the Z ring and recruits FtsW in a ^cyto^FtsL-dependent manner with FtsW recruiting FtsI. FtsN arrives, and ^cyto^FtsN interacts with FtsA in the cytoplasm, and the ^E^FtsN domain acts in the periplasm, switching both FtsA and FtsQLB from the OFF to the ON conformation, resulting in activation of FtsWI. In the model, activation occurs when the AWI (activation of FtsWI) domain in FtsL (which overlaps the CCD [constriction control domain]) becomes available to contact FtsI. Activation mutations (*) in *ftsA*, *ftsB*, *ftsL*, and *ftsW* require less FtsN. FtsB* and FtsL* are thought to switch FtsQLB to the ON state. FtsA* may also do this, whereas FtsW* is likely to lead to an enzymatically active form of FtsW. For simplicity ZipA, FtsEX, and FtsK are not depicted. FtsZ, FtsA, FtsB, FtsQ, FtsL, FtsW, FtsI, and FtsN are indicated by single letters. The red rectangles highlight interactions between FtsL and FtsWI.

Cell division in most bacteria is carried out by a large protein complex called the divisome or septal ring (1, 2). In Escherichia coli, it consists of 10 essential proteins, 2 quasi-essential proteins (FtsEX), and an ever-increasing number of nonessential proteins. The essential (and quasi-essential) proteins include FtsZ, which assembles into treadmilling filaments that are tethered to the membrane by FtsA and ZipA (Z ring), and 7 additional proteins which display the following dependency for recruitment: FtsE/X < FtsK < FtsQ < FtsL/B < FtsW < FtsI, and FtsN (Fig. 1) (1–4). Among these, FtsW is a newly described glycosyltransferase of the SEDS (septation, elongation, division, and sporulation) family that works in concert with a transpeptidase (FtsI [PBP3]) to synthesize septal peptidoglycan (PG) (5–8). A key step in cell division is the activation of these enzymes by FtsN, the last arriving essential protein (3, 9), which acts through FtsA and the FtsQLB complex (10–12).

The FtsQLB complex is widely conserved among peptidoglycan-containing bacteria and links the Z ring to the septal PG synthesis machinery (FtsWI) (13). Each protein in the FtsQLB complex is a bitopic membrane protein with a short cytoplasmic region connected to a larger periplasmic domain by a single transmembrane domain. FtsQ targets the FtsQLB complex to the Z ring in an FtsK-dependent fashion, and the cytoplasmic domain of FtsL is required to recruit FtsW (3) (Fig. 1). FtsL and FtsB form a multimer with interactions occurring between their alpha-helical transmembrane domains as well as their putative periplasmic coiled-coil domains (14–17). They also interact with FtsQ through their C-terminal domains that lie beyond the coiled-coil domains forming a 1:1:1 complex which may dimerize (13, 15, 18). The structure of a peptide corresponding to the C-terminal region of FtsB bound to the periplasmic domain of FtsQ was recently determined (19, 20).

Activation of FtsWI by FtsN requires two domains of FtsN; the ^cyto^FtsN domain acts on FtsA, and the ^E^FtsN domain, a short putative helical segment in the periplasm, likely acts on FtsQLB (10, 21, 22, 36) (Fig. 1). In a proposed model, FtsN switches both FtsA and FtsQLB to an ON state which activates FtsWI (10, 11). This regulatory model is based in part upon the isolation of “activation (superfission)” mutations (requiring less FtsN) in *ftsL* and *ftsB* which identified a short periplasmic region in both proteins, designated CCD for constriction control domain (10). The CCD connects the coiled-coil domain of each protein to its distal C-terminal region, which binds to FtsQ (13, 18). It is not clear how these mutations work, but it is likely they mimic FtsN action, resulting in a change in conformation of the FtsQLB complex to the ON state that activates FtsWI (Fig. 1). Activation mutations have also been isolated in *ftsA* and *ftsW* (10, 12). Such mutations in *ftsA* could cause it to act on FtsQLB or FtsW, whereas such mutations in *ftsW* could lead to an enzymatically active conformation. To address the mechanism of FtsWI activation, we set out to isolate dominant negative mutations in *ftsL* and *ftsB*. Such mutations should yield an FtsQLB complex that no longer activates FtsWI and yield information about the activation mechanism. By exploring the effect of the dominant negative mutations, as well as the activation mutations, on the recruitment and activation of FtsWI, we find an essential role for FtsL in the activation of FtsWI.

## RESULTS

### Isolation of dominant negative mutations in *ftsL* but not *ftsB*.

**FIG 2:**
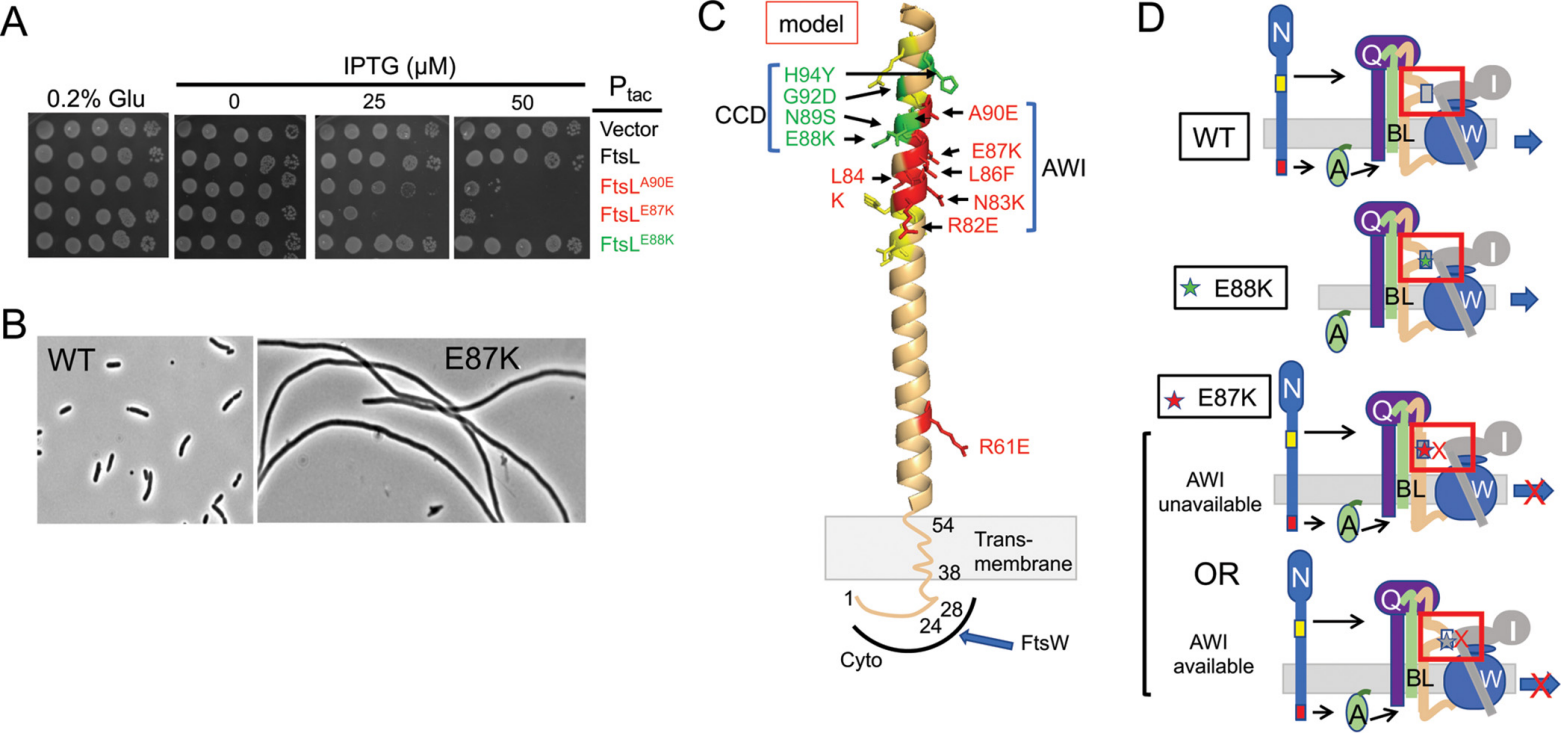
Isolation of dominant negative mutations in *ftsL*. (A) Spot test of dominant negative mutations in *ftsL*. *ftsL* was subjected to random PCR mutagenesis, cloned downstream of the *tac* promoter in an expression vector containing an IPTG-inducible promoter (pJF118EH), and transformed into JS238. Transformants were screened for sensitivity to IPTG. *ftsL^WT^* and *ftsL^E88K^* (an activation allele) were included as controls and are not toxic. Several strong dominant negative mutations (*ftsL^E87K^*, *ftsL^L86F^*, and *ftsL^A90E^*) and two weak mutations (*ftsL^R61C^* and *ftsL^E24K^*) were obtained in this way (Table 1). Additional mutations were obtained by site-directed mutagenesis. (B) Dominant negative mutants inhibit division. Phase contrast micrographs of JS238 expressing *ftsL* or *ftsL^E87K^* (derivatives of pKTP100 [P*_tac_*::*ftsL*]) grown in liquid culture and induced with 50 μM IPTG for 2 h. Induction of the other alleles also inhibited division (Table 1). (C) FtsL, residues 54 to 99, was modeled (for illustration purposes) as an alpha helix since it is thought to form a continuous alpha helix with the TM, and this region is also thought to form a coiled coil with FtsB. Altering the residues in green leads to activation mutations, whereas altering those residues in red results in dominant negative mutations. Altering the residues in yellow had no effect. Note that the activation mutations affect residues that lie mostly on one side of the helix, whereas the dominant negative mutations affect residues that lie mostly on the other side. The red residues (including L86 and E87) identify a region designated AWI (activation of FtsWI). The positions of residues 24 and 28 in the cytoplasmic domain are indicated along with the transmembrane (TM) domain. The cytoplasmic domain of FtsL is required to recruit FtsW, which in turn recruits FtsI. (D) Cartoons depicting the effect of various mutations on the activation of FtsWI according to the model. Top, FtsN action makes AWI available; middle, FtsL^E88K^ is less dependent upon FtsN as the E88K substitution makes AWI available; bottom, FtsL^E87K^ is resistant to FtsN action, and AWI does not become available or is defective in interaction with FtsWI.

To isolate dominant negative mutations in *ftsL* and *ftsB*, they were subjected to random mutagenesis, cloned into a plasmid downstream of an IPTG-inducible promoter, and introduced into a wild-type strain. Colonies were then picked, and dominant negative mutants were identified by screening for growth inhibition after streaking on plates containing increasing amounts of IPTG (isopropyl-β-d-thiogalactopyranoside). Three strong dominant negative mutations were obtained in *ftsL* (*ftsL^E87K^*, *ftsL^L86F^*, and *ftsL^A90E^*) as well as two weak mutations (*ftsL^R61C^* and *ftsL^L24K^*), but none were obtained in *fts*B (Fig. 2A and Table 1). Changing *ftsL^R61C^* to *ftsL^R61E^* resulted in a stronger dominant negative mutant (Table 1), while *ftsL^L24K^* is discussed later. Induction of the *ftsL* alleles in liquid culture resulted in filamentation (Fig. 2B and Table 1). Complementation tests confirmed they were loss of function mutations, as they were unable to complement a Δ*ftsL* strain (Fig. S1A, Table 1). Interestingly, three of these mutations overlapped the CCD domain, which was previously defined by activation mutations that decrease the dependency upon FtsN (10, 11) (Fig. 2C). Using site-directed mutagenesis, we altered additional residues around the CCD and isolated three additional dominant negative mutations (*ftsL^R82E^*, *ftsL^N83K^*, and *ftsL^L84K^*) (Fig. 2C and Table 1). However, extending the mutagenesis to flanking regions as well as the C-terminal region of *ftsL* did not yield any additional dominant negative mutations (Fig. 2C and Table 1). Although the residues we identified overlap the CCD, they are distinct from the residues involved in activation and lie mostly on the opposite side of a putative alpha helix. Since these mutations lead to a dominant negative effect, they behave as though they are nonresponsive to FtsN, just the opposite of activation mutations (Fig. 2D). We designate the region identified by the dominant negative residues as AWI (activation of FtsWI) based on the results described below.

**TABLE 1:** Summary of the point mutations in *ftsL* and *ftsB*^*a*^

Mutations	Complementation^*b*^	DN	Relative strength of DN^*c*^	Suppression of DN by E88K^*c*^	Suppression of DN by FtsN expression^*d*^
FtsL mutations					
L24K	NT	Weak	+	NT	NT
L24K, I28K	No	Yes	++	NT	NT
R61C	NT	Weak	+	NT	NT
R61E	No	Yes	++	Yes	Yes
L77K	NT	No			
D78K	NT	No			
E80K	NT	No			
W81A	NT	No			
R82E	No	Yes	+++	NT	NT
N83K	No	Yes	+++	Yes	NT
L84K	No	Yes	+++	NT	NT
L86F	No	Yes	++++	No	No
E87K	No	Yes	++++	No	No
A90E	No	Yes	+++	Yes	Yes
L91K	Yes	No			
R96E	Yes	No			
A101K	Yes	No			
L105D	Yes	No			
M107K	Yes	No			
E115K	Yes	No			
P112-Stop	Yes	No			
Q114-Stop	Yes	No			
FtsB mutations^*e*^					
N43K	Yes	No			
N50K^*f*^	No	No			
Q52K	Yes	No			
F54K	Yes	No			
I57K	Yes	No			
L60K	Yes	No			
A66K	Yes	No			

a DN, dominant negative.

b NT, not tested. Complementation and suppression tests were done in strain SD399.

c Indicates IPTG concentration that inhibited colony formation: ++++, 25 μM; +++, 30 μM; ++, 50 to 100 μM; +, cells filamentous at 100 μM. Dominant negative tests were done in JS238 with derivatives of pKTP100 (P*_tac_*::*ftsL*) and pKTP101 (P*_tac_*::*ftsB*) carrying the indicated mutations.

d Suppression by *ftsN* was done with strain SD399 (pSD256) containing plasmids pSD296 (*ftsL^m^*) and pSEB417 (*ftsN*).

e For these strains, complementation was done using strain BL155/pBL194.

f It is likely that this mutant is unstable.

10.1128/mBio.03012-20.1 FIG S1 Characterization of dominant negative mutations in *ftsL*. (A) Dominant negative mutations do not complement an Δ*ftsL* strain. Two dominant negative alleles of *ftsL* were tested for their ability to complement an *ftsL* depletion strain by transforming SD439 (*ftsL*::*kan*)/pSD296 (P_ara_::*ftsL*) with pKTP100 (P*_tac_*::*ftsL*) derivatives carrying different *ftsL* alleles. The toxicity of these alleles was tested on plates containing both arabinose (induced WT *ftsL*) and IPTG (to induce the allele to be tested) (center panel). The ability to complement the *ftsL* depletion strain was assessed on plates without arabinose or IPTG (basal level of expression from pKTP100 (P*_tac_*::*ftsL*) is sufficient for complementation) (right panel). (B) Lineup of a region (amino acids 57 to 110) of the periplasmic domain of FtsL. FtsLs from a variety of Gram-negative and Gram-positive bacteria were aligned using Cobalt. Residues in red are more conserved than residues in blue, which are more conserved than those in gray. E87 is indicated by the arrow. (C) Examination of additional substitutions at position 87 in FtsL on toxicity. Derivatives of pKT100 (P*_tac_*::*ftsL*) carrying various alleles of *ftsL* were introduced into JS238 and tested for toxicity by performing spot tests on plates containing increasing amounts of IPTG. (D) Effect of the dominant negative *ftsL* mutations on the recruitment of GFP-FtsI. SD285 (*leu*::Tn*10* P*_206_*::*gfp-ftsI*) was transformed with pSD296 (P*_ara_*::*ftsL*) derivatives expressing *ftsL^E87K^*, *ftsL^A90E^*, or *ftsL*. Three hours after induction with 0.2% arabinose and 25 mM IPTG, samples were taken and examined by phase and fluorescent microscopy. Download FIG S1, TIF file, 9.03 MB. Copyright © 2020 Park et al. 2020 Park et al. This content is distributed under the terms of the Creative Commons Attribution 4.0 International license.

Of residues composing the CCD domain of FtsL, residue E88 is the most conserved, and mutational analysis indicated that loss of the negative charge results in the activation phenotype (10). The neighboring residue E87 is even more conserved (Fig. S1B) and was altered in one of our dominant negative mutants. Additional analysis indicates that changing this residue to amino acids other than aspartate produces a dominant negative phenotype (Fig. S1C). Thus, the loss of the negative charge in two neighboring glutamate residues yields contrasting phenotypes. Since loss of the negative charge in each case produced their respective phenotypes, it strongly suggests that these mutations disrupt rather than enhance interactions.

In our random mutagenesis screen, we did not isolate dominant negative mutations in *ftsB*; however, since six of the dominant negative mutations in *ftsL* overlapped the CCD, we used site-directed mutagenesis to alter the more conserved residues that overlap FtsB’s CCD domain. Seven residues flanking the CCD domain were altered, but none produced a dominant negative phenotype (Table 1). Six of these still complemented an *ftsB* deletion strain. This result suggests that the dominant negative mutations are unique to *ftsL*.

### Dominant negative FtsL mutants are defective in activation of septal PG synthesis.

A dominant negative phenotype could result from incorporation of an FtsL mutant into the FtsQLB complex that fails to (i) recruit downstream proteins (FtsWI), (ii) respond to FtsN (FtsQLB locked in OFF state), or (iii) generate an output signal in response to FtsN (ON state but failure to interact with a downstream partner). To test the first possibility, we assessed the localization of green fluorescent protein (GFP)-FtsI, which depends upon FtsW (3, 23). It was present in crossbands within filamentous cells following expression of *ftsL^E87K^* or *ftsL^A90E^*, indicating recruitment to the Z ring (Fig. S1D). This result suggests that the *ftsL* mutations blocked either the response to FtsN or a downstream event such as interaction with FtsWI.

The dominant negative *ftsL* mutations were tested to see if they could be rescued by a strong activation mutation (*ftsL^E88K^*) in *cis*. While *ftsL^R61E^* and *ftsL^A90E^* were readily rescued by *ftsL^E88K^*, *ftsL^L86F^* and *ftsL^E87K^* were not (Fig. S2A). If we assume that *ftsL^E88K^* mimics FtsN action and switches FtsQLB to the ON state, it suggests that *ftsL^R61E^* and *ftsL^A90E^* are able to carry out steps downstream of FtsN action. Based on these results, we suspected overexpression of *ftsN* would also rescue *ftsL^A90E^* and *ftsL^R61E^* but not *ftsL^E87K^* or *ftsL^L86F^*. This, in fact, was the case (Fig. S2B and Table 1). Since *ftsL^R61E^* and *ftsL^A90E^* were rescued by enhancing the activation signal (by introducing an *ftsL* activation mutation or *ftsN* overexpression), it suggests they favor the OFF state (partially resistant to FtsN) but can carry out downstream events when activated. We therefore focused on *ftsL^L86F^* and *ftsL^E87K^* since it is unclear if they are locked in the OFF state or are unable to produce a signal in response to FtsN.

10.1128/mBio.03012-20.2 FIG S2 Test for rescue of dominant negative *ftsL* mutations by an *ftsL* activation mutation or by *ftsN* overexpression. (A) Rescue by an *ftsL* activation mutation in *cis*. The *ftsL^E88K^* mutation was added to various *ftsL* alleles in *cis* and then tested for complementation. To do this, SD439 (*ftsL*::*kan*/pSD296 [P*_ara_*::*ftsL*]) was transformed with derivatives of pKTP100 (P_tac_::*ftsL*) carrying the various mutations. The strains were spotted on plates at 30°C without arabinose to deplete WT *ftsL*, and IPTG was added to induce the various *ftsL* alleles. *ftsL^A90E^* and *ftsL^R61E^* were rescued, but *ftsL^E87K^* and *ftsL^L86F^* (Table 1) were not. (B) Rescue by overexpression of *ftsN*. To test if *ftsN* overexpression can rescue any of the dominant negative alleles of *ftsL*, SD399 (*ftsL*::*kan*/pSD256 [*repA*^TS^ P_syn135_::*ftsL*]) containing plasmids expressing the *ftsL* alleles under an arabinose inducible promoter (derivatives of pSD296 [P_ara_::*ftsL*]) was transformed with a plasmid with *ftsN* under IPTG control (pSEB417 [P_204_::*ftsN*]). The strains were spotted at 37°C to deplete WT *ftsL*, and arabinose was added to induce the *ftsL* allele, and IPTG added to induce *ftsN*. Download FIG S2, TIF file, 10.6 MB. Copyright © 2020 Park et al. 2020 Park et al. This content is distributed under the terms of the Creative Commons Attribution 4.0 International license.

### Dominant negative FtsL mutants are rescued by FtsW activation mutants.

Based on our results, we hypothesized that activation of FtsWI requires a signal from the periplasmic domain of FtsL (AWI domain) which is made available by FtsN action or *ftsL* activation mutations. We also hypothesized that activated alleles of *ftsW* might rescue a strong dominant negative *ftsL* allele since they require less input from FtsN. Two such *ftsW* alleles exist: *ftsW^M269I^*, which weakly bypasses *ftsN* (12), and *ftsW^E289G^*, which was isolated as described in Materials and Methods and bypasses *ftsN*. The latter mutation was also isolated using another approach and shown to bypass *ftsN* (24).

**FIG 3:**
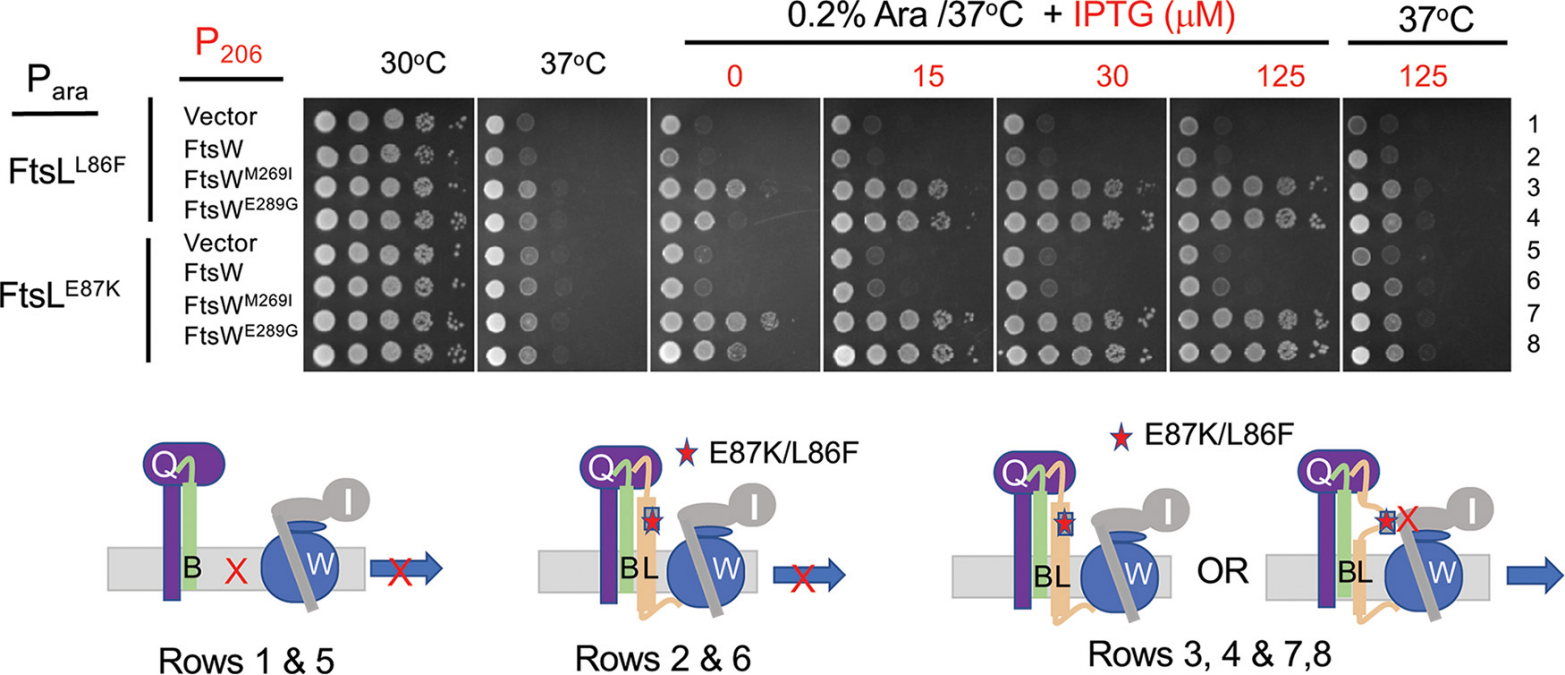
Rescue of dominant negative mutations in *ftsL* by overexpression of active FtsW mutants. SD399 (*ftsL*::*kan*/pSD256 [*repA*^ts^::*ftsL*]) containing derivatives of pSD296 (P*_ara_*::*ftsL*) with different alleles of *ftsL* was transformed with derivatives of pSEB429 (P_204_::*ftsW*) carrying WT *ftsW* or either of two active alleles of *ftsW*. Transformants were spot tested at 37°C (to deplete WT FtsL) in the presence of arabinose (to induce the *ftsL* allele present on derivatives of pSD296) and increasing concentrations of IPTG to induce alleles of *ftsW* (*ftsW*, *ftsW^M269I^* or *ftsW^E289G^*). The cartoons below depict the interpretation of the results. On the left, FtsWI is not recruited in the absence of FtsL; center, FtsWI is recruited but not activated in the presence of a dominant negative FtsL mutant; right, active FtsW mutants suppress dominant negative FtsL mutants in one of two ways (see the text).

To see if these *ftsW* alleles could rescue f*tsL^L86F^* or *ftsL^E87K^*, a plasmid with these alleles under an arabinose-inducible promoter (derivatives of pSD296 [P*_ara_*::*ftsL*]), as well as a compatible plasmid with *ftsW* alleles under an IPTG-inducible promoter (derivatives of pSEB429 [P*_204_*::*ftsW*]), were introduced into SD399 (*ftsL*::*kan*/pSD256 [*repA*^ts^::*ftsL*]). The resultant strains were tested on plates at 37°C to deplete wild-type (WT) *ftsL*, and arabinose and IPTG were added to induce the *ftsL* and *ftsW* alleles, respectively. Expression of *ftsW^M269I^* and *ftsW^E289G^*, but not *ftsW*, rescued the dominant negative *ftsL* alleles (Fig. 3). These *ftsW* activation alleles still required the presence of *ftsL*, as they could not bypass it (Fig. 3, right panel). Also, *ftsW^M269I^* was able to rescue an allele containing both mutations (*ftsL^L86F^*^/^*^E87K^*), whereas overexpression of *ftsN* could not (Fig. S3A). These results indicate that *ftsL^L86F^*^/^*^E87K^* cannot transmit the periplasmic signal in response to FtsN.

10.1128/mBio.03012-20.3 FIG S3 Effect of activation mutations in *ftsW* and *ftsB* as well as *ftsN* overexpression on the rescue of dominant negative *ftsL* mutations. (A) Overexpression of an activation allele of *ftsW*, but not *ftsN*, suppresses a dominant negative allele of *ftsL (ftsL^L86F/E87K^)*. SD399 (*ftsL*::*kan*/pSD256 [*repA*^ts^-*ftsL*]) was transformed with plasmids expressing either *ftsL* or a dominant negative allele of *ftsL* (*ftsL^E87K^*^/L^*^86F^*) under an arabinose-inducible promoter (derivatives of pSD296 [P_ara_::*ftsL*]) and plasmids expressing *ftsW^M269I^* or *ftsN* under an IPTG-inducible promoter (pSEB429-I [P_204_::*ftsW^M269I^*] and pSEB417 [P_204_::*ftsN*], respectively). The strains were spotted on plates at 37°C (to deplete WT *ftsL*) containing 0.2% arabinose to induce the *ftsL* alleles, with increasing concentrations of IPTG to induce *ftsW^M269I^* or *ftsN*. The second panel lacks arabinose, demonstrating that the *ftsW* activation mutations cannot bypass *ftsL.* (B) An activated allele of *ftsB* cannot suppress a dominant negative *ftsL* mutation. Strain PK168-1 (*ftsB^E56A^ recA*::*aadA ftsL*::*kan*/pSD296 [P_ara_::*ftsL*]) containing pKTP100 (P*_tac_*::*ftsL*) or a version expressing *ftsL^E87K^* was subjected to spot tests on plates without arabinose to deplete WT *ftsL* and with IPTG added to induce the *ftsL* allele cloned in derivatives of pKTP100. Download FIG S3, TIF file, 10.6 MB. Copyright © 2020 Park et al. 2020 Park et al. This content is distributed under the terms of the Creative Commons Attribution 4.0 International license.

Although the above-described results demonstrate that the two dominant negative mutations (*ftsL^L86F^* or *ftsL^E87K^*, alone or combined) block FtsN, they do not distinguish between whether they lock FtsQLB in the OFF state (nonresponsive to FtsN) or prevent a downstream step (responsive to FtsN but failing to interact with FtsWI). We suspect the latter for the following reasons. To rescue *ftsL^L86F^* or *ftsL^E87K^*, *ftsW^E289G^* has to be overexpressed, whereas the chromosomal level of *ftsW^E289G^* was sufficient to bypass *ftsN* (expression of *ftsW* or the activation alleles from the plasmids complement an *ftsW* depletion mutant in the absence of IPTG [Fig. S4A], whereas 15 to 30 μM is required to rescue *ftsL^L86F^* or *ftsL^E87K^*). Consistent with this, expression of *ftsL^E87K^* is toxic to a strain with *ftsW^M269I^* on the chromosome (Fig. S4B), highlighting that an active *ftsW* allele cannot bypass the dominant negative *ftsL* mutation at the chromosomal level. These results suggest that the dominant negative *ftsL* mutants are defective in interaction with FtsWI in the periplasm (lack of the periplasmic interaction necessitates overexpression of an active *ftsW*). Consistent with the *ftsL* mutations blocking a step downstream of FtsN action, an active *ftsB* mutation, *ftsB^E56A^*, which can also bypass *ftsN* (10), cannot suppress *ftsL^E87K^* (Fig. S3B). This result is also consistent with an activation mutation in *ftsL* or overexpression of *ftsN* being unable to rescue *ftsL^E87K^* (Fig. S3A). Furthermore, all substitutions in *ftsL^E87^* that remove the negative charge are dominant negative (Fig. S1C), suggesting they disrupt, rather than enhance, an interaction. Therefore, we favor the idea that these mutations in the AWI domain abrogate FtsL’s interaction with FtsWI and that under physiological conditions, FtsWI is recruited by ^cyto^FtsL and activated by FtsQLB when it is in the ON state (AWI available).

10.1128/mBio.03012-20.4 FIG S4 Effect of activation mutations in *ftsW* on complementation and rescue of a dominant negative *ftsL* allele. (A) Test of *ftsW* alleles for complementation of an *ftsW* depletion strain in the absence of IPTG. To test if the addition of IPTG was required for complementation of an *ftsW* depletion strain, EC912 (*ftsW*::*kan*/pDSW406 [P_ara_::*ftsW*]) was transformed with derivatives of pSEB429 (P_204_::*ftsW*) carrying various alleles of *ftsW*. The strains were spot tested on plates without arabinose and with or without 200 μM IPTG. (B) Expression of *ftsL^E87K^* inhibits a strain with the *ftsW^M269I^* mutation on the chromosome. Strains KTP1 (*ftsW^+^*) and SD247-1 (*ftsW^M269I^*) were transformed with pKTP100 (P_tac_::*ftsL*) or a derivative expressing *ftsL^E87K^*. The strains were spot tested on plates containing increasing concentrations of IPTG to test for toxicity. The strain containing the *ftsW^M269I^* is more resistant but still sensitive to *ftsL^E87K^*. Download FIG S4, TIF file, 10.6 MB. Copyright © 2020 Park et al. 2020 Park et al. This content is distributed under the terms of the Creative Commons Attribution 4.0 International license.

### Loss of ^cyto^FtsL function rescued by activation mutations in the CCD domain of FtsL.

One mutation from the random mutagenesis screen altered a residue in the ^cyto^FtsL domain (*ftsL^L24K^*). Although weak, adding a second mutation that altered a conserved residue in this domain (*ftsL^I28K^*) yielded a stronger dominant negative phenotype (Fig. S5A). Since ^cyto^FtsL is required for FtsW recruitment (13), it suggests that FtsL^L24K^, FtsL^I28K^, and the double mutant assemble into a complex with FtsQ and FtsB that poorly recruits FtsW. Consistent with this, deletion of the cytoplasmic domain of FtsL (FtsL^Δ1-30^) produced a strong dominant negative phenotype (Fig. S5B) resulting in filamentation and a failure to recruit FtsI (Fig. S5C).

10.1128/mBio.03012-20.5 FIG S5 Mutations in the cytoplasmic domain of *ftsL* result in a dominant negative phenotype and fail to recruit GFP-FtsI. (A) Mutations (L24K/I28K) in the *^cyto^ftsL* domain lead to a dominant negative phenotype. JS238 containing pKTP100 (P*_tac_*::*ftsL*) derivatives expressing different alleles of *ftsL* were tested for toxicity following induction with IPTG. (B) Deletion of the cytoplasmic domain of FtsL (*ftsL*^Δ1-30^) results in a dominant negative allele. JS238 containing pKTP104 (P_T5_::*ftsL*) or pKTP105 (P_T5_::*ftsL*^Δ1-30^) was tested for toxicity following induction with IPTG. (C) FtsL^L24K/I28K^ fails to recruit GFP-FtsI. SD285 (*leu*::Tn*10* P*_206_*::*gfp-ftsI*) was transformed with pSD296 (P*_ara_*::*ftsL^L24/I28K^*). Three hours after induction with 0.2% arabinose and 25 mM IPTG, samples were taken and examined by phase and fluorescent microscopy. Download FIG S5, TIF file, 10.6 MB. Copyright © 2020 Park et al. 2020 Park et al. This content is distributed under the terms of the Creative Commons Attribution 4.0 International license.

**FIG 4:**
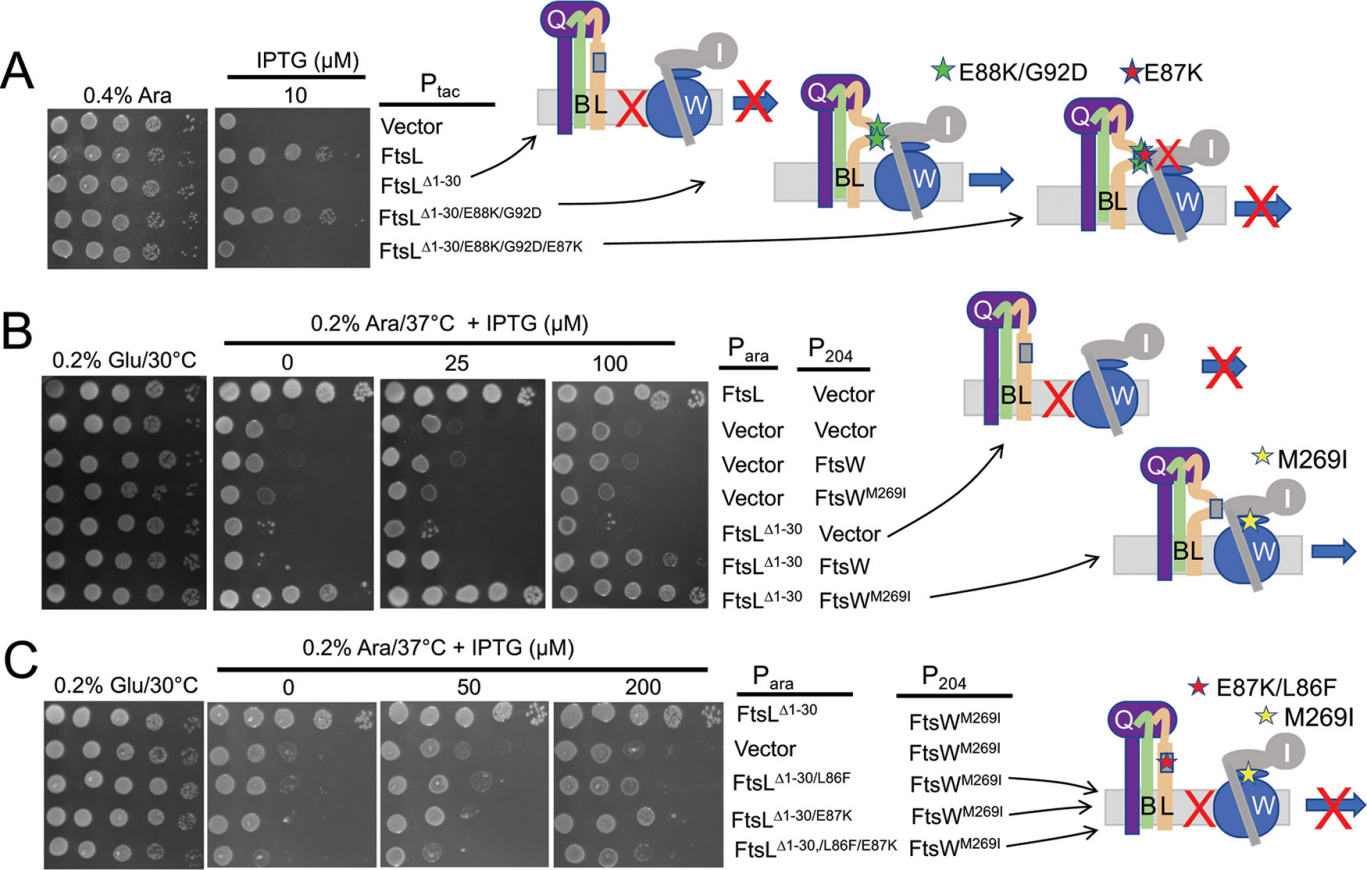
Effect of *ftsL* activation and dominant negative mutations on the rescue of FtsL^Δ1-30^. (A) *ftsL^Δ1-30^* is rescued by *ftsL* activation mutations, which is negated by an *ftsL* dominant negative mutation. SD439 (*ftsL*::*kan*/pSD296 [P*_ara_*::*ftsL*]) was transformed with derivatives of pKTP105 (*P_T5_*::*ftsL*) carrying various alleles of *ftsL* inducible with IPTG. The strains were spotted on plates without arabinose (to deplete WT *ftsL*) but with IPTG (to induce the various alleles of *ftsL* present in derivatives of pKTP105). The cartoons on the right depict the interpretation of the results. (B) Overexpression of *ftsW^M269I^* rescues *ftsL^Δ^*^1-30^. SD399 (*ftsL*::*kan*/pSD256 [*repA*^TS^ P_syn135_::*ftsL*]) carrying pKTP107 (P_ara_::*ftsL^Δ^*^1-30^) was transformed with compatible plasmids expressing different alleles of *ftsW* (derivatives of pSEB429 [P_204_::*ftsW*]) under the control of an IPTG-inducible promoter. Transformants were spotted on plates at 37°C (to deplete WT *ftsL*) in the presence of 0.2% arabinose (to induce *ftsL* alleles contained on the plasmids) and increasing concentrations of IPTG (to induce *ftsW* alleles). The cartoon indicates that FtsW^M269I^ is recruited by FtsL*^Δ^*^1-30^. (C) Dominant negative *ftsL* mutations negate rescue of *ftsL^Δ^*^1-30^ by *ftsW^M269I^*. Strain SD399 (*ftsL*::*kan*/pSD256 [*repA*^TS^ P_syn135_::*ftsL*]) was transformed with a plasmid (derivatives of pKTP107 [P_ara_::*ftsL^Δ1-30^*]) with *ftsL^Δ1-30^* under arabinose promoter control and a compatible plasmid (pSEB429 [P_204_::*ftsW*]) that carries *ftsW* or *ftsW^M269I^* under the control of an IPTG-inducible promoter. The presence of a dominant negative *ftsL* mutation negates rescue by the activated FtsW.

Since FtsN is proposed to switch FtsQLB to the ON state to activate FtsWI (10, 11), we speculated above that this switch involves a conformational change that exposes AWI to activate FtsWI. If this is the case, the activation mutations may compensate for the loss of ^cyto^FtsL by making the AWI domain available, which recruits FtsWI as well as activating it. As expected, *ftsL*^Δ1-30^ failed to complement Δ*ftsL*; however, *ftsL*^Δ1-30^ carrying two activation mutations (*ftsL^G92D^* and *ftsL^E88K^*) restored colony formation, indicating that both recruitment and activation of FtsW were restored (Fig. 4A). Further tests showed that both activation mutations were required for rescue (Fig. S6A). The rescue was fairly effective, as the average cell length of the strain expressing *ftsL*^Δ1-30/^*^G92D/E88K^* was only twice that of a strain expressing *ftsL* (Fig. S6B), whereas the strain expressing *ftsL*^Δ1-30^ was extremely filamentous. These two activation mutations also eliminated the toxicity of the *ftsL^L24K/I28K^* allele (Fig. S6C) and rescued its ability to complement (Fig. S6D). These results are consistent with a model in which the *ftsL* activation mutations cause a conformational change in FtsQLB that makes AWI available to recruit and activate FtsWI. It follows that under physiological conditions, the arrival of FtsN results in the exposure of ^AWI^FtsL, which cooperates with ^cyto^FtsL to recruit and activate FtsWI.

10.1128/mBio.03012-20.6 FIG S6 Rescue of *ftsL* cytoplasmic mutations by *ftsL* activation mutations and *ftsN* overexpression. (A) Rescue of *ftsL*^Δ1-30^ requires two *ftsL* activation mutations. SD439 (*ftsL*::*kan*/pSD296 [P*_ara_*::*ftsL*]) carrying pKTP105 (P_T5_::*ftsL*^Δ1-30^) derivatives expressing various alleles of *ftsL* were tested for their ability to complement an *ftsL* depletion strain. Complementation was tested by spotting strains on plates without arabinose (to deplete WT *ftsL*) and with IPTG to induce the various *ftsL* alleles. Loss of a functional ^cyto^FtsL domain (and therefore the ability to recruit FtsW) is compensated for by the two activation mutations *ftsL^E88K/G92D^*. The cartoon depicts the defect caused by *ftsL*^Δ1-30^ and its rescue by the activation mutations. (B) Morphology of *ΔftsL* cells rescued by *ftsL*^Δ1-30^ carrying activation mutations. SD399 (*ftsL*::*kan*/pSD256 [P_syn135_::*ftsL*]) carrying derivatives of pKTP107 [P_ara_::*ftsL*] expressing various alleles of *ftsL*^Δ1-30^ were grown to the exponential phase at 30°C with antibiotics, centrifuged, washed, and resuspended in LB with 0.2% arabinose and antibiotics at 37°C. Samples were taken 2.5 h later for photography, and the cell length distributions were determined. (C) *ftsL* activation mutations reduce the toxicity of *ftsL^L24K/I28K^*. JS238 carrying derivatives of pKTP100 (P*_tac_*::*ftsL*) expressing different alleles of *ftsL* was tested for toxicity by spotting on plates containing 200 μM IPTG to induce the *ftsL* alleles. (D) *ftsL* activation mutations rescue *ftsL^L24K/I28K^* for complementation. SD439 (*ftsL*::*kan*/pSD296 [P_ara_::*ftsL*]) containing derivatives of pKTP100 [P*_tac_*::*ftsL*] with various *ftsL* alleles was spotted on plates without arabinose (to deplete WT *ftsL*) and with IPTG to induce the mutant *ftsL* alleles. (E) Overexpression of *ftsN* suppresses *ftsL*^Δ1-30^. SD439 (*ftsL*::*kan*/pSD296 [P*_ara_*::*ftsL]*) containing derivatives of pKTP105 (P_T5_::*ftsL*^Δ1-30^) with various alleles of *ftsL* was transformed with pBL154 (*repA*^TS^ P*_syn135_*::*ftsN*) which constitutively expresses *ftsN*. Rescue of FtsL^Δ1-30^ was tested by spotting transformants on plates at 30°C without arabinose and with IPTG. The cartoon depicts that FtsN overexpression rescues FtsL^Δcyto^. (F) *ftsL* mutations do not affect interaction with FtsQ. To ensure that the *ftsL* mutations did not affect the stability of FtsL, we tested their effect on the FtsL-FtsQ interaction in the BACTH system. Strain DHM1 was transformed with plasmids carrying various alleles of *ftsL* (pUT18C derivatives) and a plasmid expressing *ftsQ* (pKT25-ftsQ). Three transformants were picked for each pair of constructs and spot tested. Column 1, FtsL; column 2, vector; column 3, FtsL^Δcyto^; column 4, FtsL^Δcyto/E88K/G92D^; column 5, FtsL^Δcyto/E88K/G92D/E87K^. (G) Interaction between FtsL and FtsWI assessed with the BACTH system. This test is similar to the test in Fig. 7 except that FtsL^Δcyto^ was replaced with FtsL^L24K/I28K^. The *ftsL* alleles were contained in pUT18C, and the *ftsW* and *ftsI* alleles were in pKT25. The plates were photographed after overnight incubation. Column 1, FtsL versus FtsI; column 2, vector versus FtsI; column 3, FtsL^L24K/I28K^ versus FtsW; column 4, FtsL^L24K/I28K^ versus FtsWM269I; column 5, FtsL^L24K/I28K^ versus FtsI; column 6, FtsL^L24K/I28K/E88K/G92D^ versus FtsW; column 7, FtsL^L24K/I28K/E88K/G92D^ versus FtsW^M269I^; and column 8, FtsL^L24K/I28K/E88K/G92D^ versus FtsI. Download FIG S6, TIF file, 10.6 MB. Copyright © 2020 Park et al. 2020 Park et al. This content is distributed under the terms of the Creative Commons Attribution 4.0 International license.

Since the *ftsL* activation mutations appear to mimic FtsN action, we expected that overexpression of *ftsN* would also rescue *ftsL*^Δ1-30^. To test this, an *ftsL* depletion strain was transformed with a plasmid expressing *ftsL*^Δ1-30^ and a plasmid that overexpresses *ftsN* to a level that is sufficient to bypass *zipA* or *ftsEX* (21). The increased FtsN rescued *ftsL*^Δ1-30^ (Fig. S6E), suggesting that the excess FtsN caused AWI to be available to recruit and activate FtsWI, indicating that overexpression of *ftsN* is comparable to combining the two activation mutations (*ftsL^G92D^* and *ftsL^E88K^*) in rescuing *ftsL*^Δ1-30^.

### Dominant negative *ftsL* mutations negate rescue by activation mutations.

If *ftsL* activation mutations rescue *ftsL*^Δ1-30^ by making AWI available to recruit and activate FtsWI, the dominant negative mutations should impair rescue by blocking the interaction. As seen in Fig. 4A, addition of *ftsL^E87K^* negated the rescue of *ftsL*^Δ1-30^ by the activation mutations, consistent with *ftsL^E87K^* blocking interaction between the AWI domain and FtsWI.

The FtsQLB complex probably exists in equilibrium between ON and OFF states, with the activation mutations and overexpression of FtsN favoring the ON state (AWI available). Overexpression of FtsW or FtsW^M269I^ may also tip the equilibrium to the ON state and rescue *ftsL*^Δ1-30^, as the increased level of FtsW may promote capture of the ON state. Indeed, expression of *ftsW^M269I^*, even at low levels of induction, rescued *ftsL*^Δ1-30^, and at higher levels of induction, WT *ftsW* also started to rescue (Fig. 4B).

Earlier, we showed that overexpression of *ftsW^M269I^* and *ftsW^E289G^*, but not *ftsW*, rescued *ftsL* carrying dominant negative mutations (Fig. 3). This result is consistent with these activated mutants being recruited by the FtsL mutants (through ^cyto^FtsL) but not requiring an activation signal from the AWI domain (via FtsN) (12). In the absence of ^cyto^FtsL, however, our results suggest rescue requires a functional AWI in FtsL^peri^. If so, the dominant negative mutations should be detrimental in this context. As expected, the addition of either of two dominant negative mutations (*ftsL^L86F^* or *ftsL^E87K^*) to *ftsL*^Δ1-30^ prevented rescue by FtsW^M269^*^I^* (Fig. 4C). These results are consistent with AWI being required to recruit FtsWI in the absence of ^cyto^FtsL. It is worth noting that when either of two FtsL domains is nonfunctional (due to either inactivation of the cytoplasmic domain or the presence of the dominant negative mutations [such as L86F and E87K] in full-length FtsL), the active FtsW mutants must be overexpressed to rescue growth (see Discussion).

### Rescue of FtsL^Δ1-30^ by overexpression of FtsI.

In the hierarchical assembly pathway, FtsW is recruited in a ^cyto^FtsL-dependent manner followed by FtsI, which is recruited by interaction between FtsW and the transmembrane segment of FtsI (23). However, we considered the possibility that with FtsL^Δ1-30^, the recruitment is reversed or FtsWI is recruited as a complex through interaction of AWI with FtsI. This thinking was driven in part by geometric constraints. The periplasmic domain of FtsL is thought to be a continuous alpha helix with its transmembrane domain such that the AWI domain would extend about ∼45 Å away from the cytoplasmic membrane (15) (Fig. S7). In the RodA-PBP2 structure (homologous to FtsW-FtsI), the non-penicillin-binding (nPB) or pedestal domain of PBP2 sits on top of RodA and extends into the periplasm (25). Assuming FtsW-FtsI adopts a similar structure, FtsI could contact AWI in FtsL.

10.1128/mBio.03012-20.7 FIG S7 Diagram indicating the position AWI of FtsL relative to the membrane and the RodA-PBP2 complex. Part of the periplasmic domain of FtsL was modeled as an alpha helix (residues 57 to 99) and positioned next to the structure of the RodA-PBP2 complex (PDB ID: 6PL6). The RodA-PBP2 complex is homologous to the FtsW-PBP3 (FtsI) complex. The non-penicillin binding domain (nPB) and penicillin binding domain (PD) are indicated. Key FtsL residues (E87 [AWI], E88 [CCD]) are about 4.3 nm from the membrane. The position of R61 is also indicated. Download FIG S7, TIF file, 10.6 MB. Copyright © 2020 Park et al. 2020 Park et al. This content is distributed under the terms of the Creative Commons Attribution 4.0 International license.

**FIG 5:**
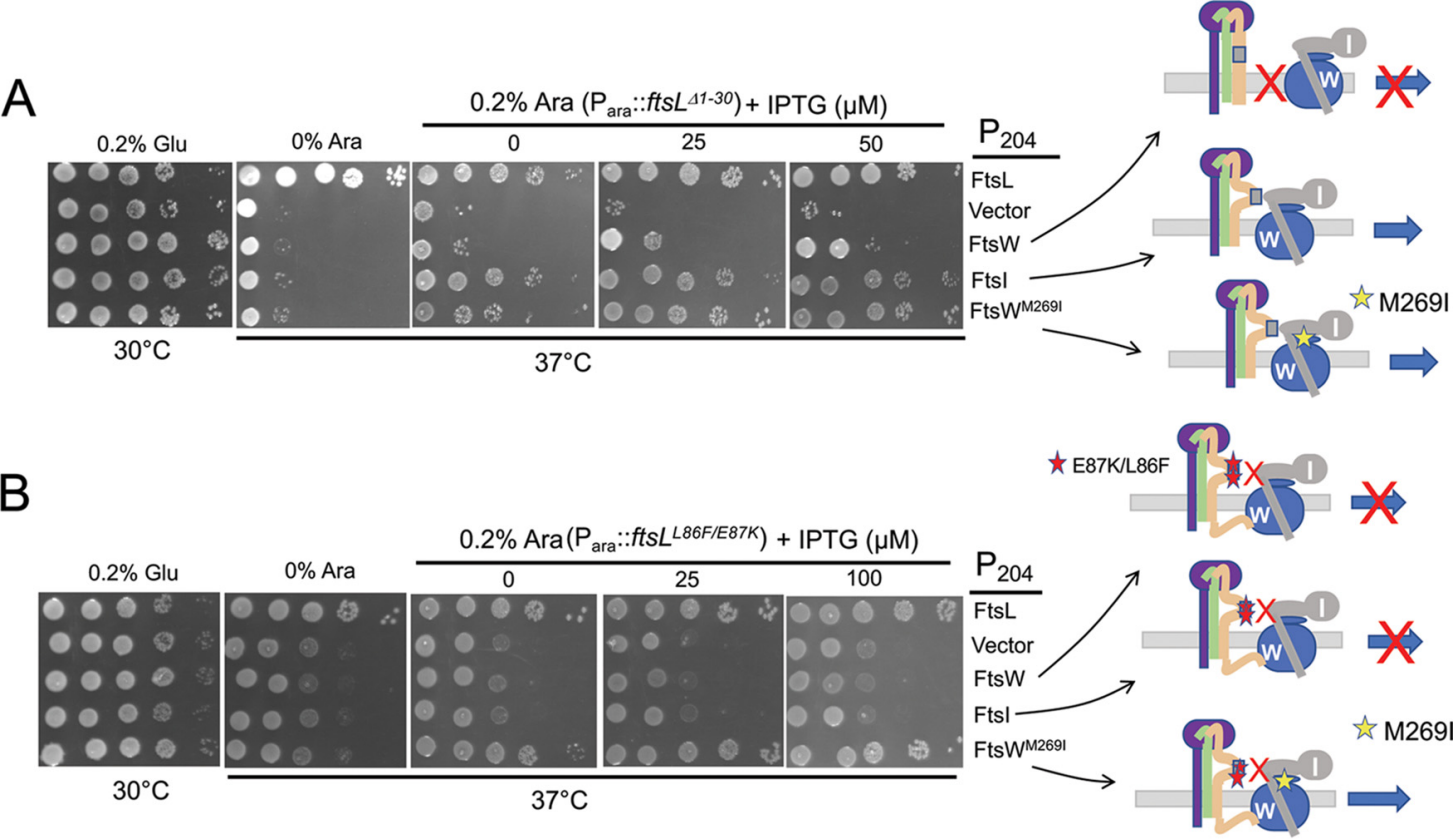
Rescue of FtsL*^Δ^*^1-30^ by *ftsI* expression. (A) *ftsI* expression rescues FtsL*^Δ^*^1-30^. To test if overexpression of *ftsI* could rescue FtsL*^Δ^*^1-30^, PK4-1 (*ftsL*::*kan*/pKTP108 [*repA^TS^*::*ftsL*]) was transformed with a plasmid expressing *ftsL^Δ^*^1-30^ (pKTP107X/P_ara_::*ftsL^Δ^*^1-30^-*6Xhis*) and a plasmid expressing *ftsI* (pSEB420/P_204_::*ftsI*) or *ftsW* (pSEB429/P_204_::*ftsW*) under an IPTG-inducible promoter. The strains were spot tested on plates at 37°C to deplete *ftsL*. In addition, arabinose was added to induce *ftsL^Δ^*^1-30^, and increasing concentrations of IPTG were added to induce *ftsI* or *ftsW*. (B) *ftsI* overexpression cannot rescue a dominant negative *ftsL* allele. SD399 (*ftsL*::*kan*/pSD256 [*repA*^ts^::*ftsL*]) was transformed with pSD296-2 (P_ara_::*ftsL^L86F/E87K^*) and pSEB420 (P_204_::*ftsI*) or pSEB429 (P_204_::*ftsW*). Transformants were spot tested at 37°C (to deplete *ftsL*) on plates containing 0.2% arabinose (to induce *ftsL^L86F/E87K^*) and increasing concentrations of IPTG (to induce *ftsI* or *ftsW*).

If FtsI interacts with the AWI domain, overexpression of *ftsI* may rescue FtsL^Δ1-30^ by enhancing the interaction with ^AWI^FtsL and shifting the equilibrium of FtsQLB from OFF to ON through mass action. To test this, we compared the ability of the overexpression of *ftsI* and *ftsW* to rescue FtsL^Δ1-30^. As shown in Fig. 5A, expression of *ftsI* was much more efficient than that of *ftsW* in rescuing FtsL^Δ1-30^. The efficient rescue of FtsL^Δ1-30^ by FtsI suggests that it captures the transient ON state of FtsQLB (AWI exposed) and converts FtsQL^Δ1-30^B into an active form similar to *ftsL* activation mutations (Fig. 4A). The rescue of FtsL^Δ1-30^ by overexpression of FtsW may involve the formation of an FtsWI complex that interacts with AWI, and the more efficient rescue of FtsL^Δ1-30^ by activated FtsW (compared to WT FtsW seen in Fig. 4B) may be due to it being active and more readily forming a complex with FtsI.

The above-described results indicate that the signal from FtsN via the AWI domain goes through FtsI. As shown earlier, expression of activated alleles of *ftsW* suppressed *ftsL^L86F^* or *ftsL^E87K^*, as they no longer require the signal from AWI. In contrast, WT *ftsW* cannot suppress these alleles, as it still requires the AWI activation signal. Likewise, overexpression of *ftsI* would not be expected to rescue FtsL carrying the dominant negative *ftsL* mutations since the AWI activation signal would not be present. As expected, overexpression of *ftsI* was unable to suppress *ftsL^L86F^*^/^*^E87K^*, indicating the AWI signal was still required (Fig. 5B).

**FIG 6:**
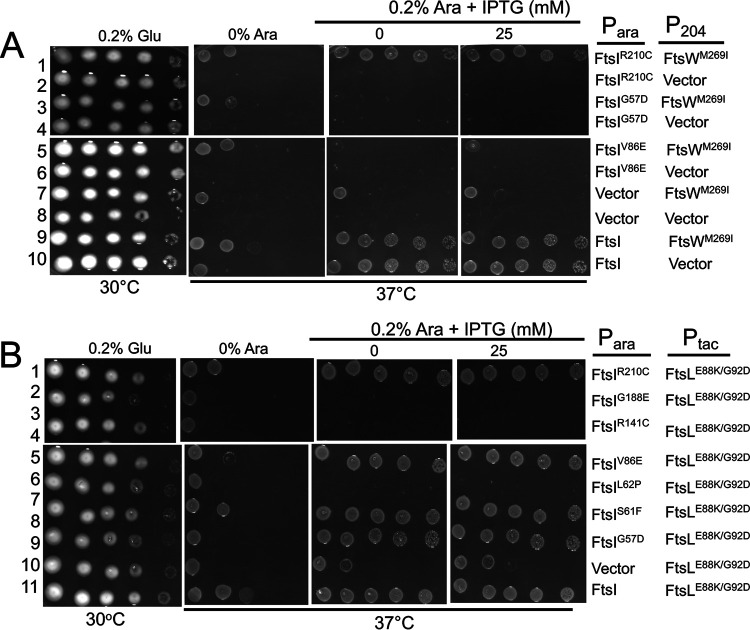
Rescue of FtsI mutants by activated FtsL and FtsW mutants. (A) Rescue of FtsI mutants by FtsW^M269I^. To test if the FtsI mutants could be rescued by an activated allele of *ftsW*, MCI23 (*ftsI23^ts^ recA*::*spc*) was transformed with compatible plasmids expressing an activated allele of *ftsW* (pSEB429 [P*_204_*::*ftsW^M269I^*]) and *ftsI* alleles under arabinose promoter control (derivatives of pKTP109 [P_ara_::*ftsI*]). Transformants were spot tested on plates at 37°C (to inactivate *ftsI23*^ts^) with arabinose added to induce the *ftsI* alleles and increasing concentrations of IPTG to induce *ftsW^M269I^*. Note: additional alleles of *ftsI* were not rescued by *ftsW^M269I^* (Fig. S8). (B) Rescue of FtsI mutants by *ftsL^E88K/G92D^*. To test rescue of FtsI mutants by activated FtsL, MCI23 (*ftsI23^ts^ recA*::*spc)* was transformed with compatible plasmids expressing an activated allele of *ftsL* (pKTP100* [P*_tac_*::*ftsL^E88K/G92D^*]) and the various *ftsI* alleles under arabinose promoter control (derivatives of pKTP109 [P_ara_::*ftsI*]). Transformants were spot tested on plates at 37°C (to inactivate *ftsI23*^ts^), and arabinose was added to induce the *ftsI* alleles, and increasing concentrations of IPTG were added to induce *ftsL^E88K/G92D^*.

The possibility that AWI recruits and activates FtsWI by acting through FtsI was further examined by testing FtsI mutants isolated by the Weiss lab (26). These mutants localize to the division site but fail to complement a depletion strain and recruit FtsN. We reasoned that if an active FtsL acts directly on FtsW (to generate an active FtsW), an activated FtsL should have no more ability to rescue such mutants than an active FtsW mutant. However, if an activated FtsL acts on FtsI, it might have more ability to rescue FtsI mutants than an active FtsW. Therefore, each FtsI mutant was tested to see if it could be rescued by an active form of FtsL or FtsW (FtsL^G92D/E88K^ and FtsW^M269I^, respectively). Of the seven FtsI mutants tested, two mutants (FtsI^S61F^ and FtsI^R210C^) were rescued by both FtsW^M269I^ and FtsL^G92D/E88K^ (Fig. 6 and Fig. S8). However, FtsL^G92D/E88K^ rescued two additional mutants (FtsI^G57D^ and FtsI^V86E^; Fig. 6B, rows 5 and 9) not rescued by FtsW^M269I^ (Fig. 6A, rows 3 and 5). The rescue of these two mutants by an activated FtsL (but not an activated FtsW) suggests that AWI acts through FtsI to activate FtsW rather than acting directly on FtsW.

10.1128/mBio.03012-20.8 FIG S8 Testing the rescue of additional alleles of *ftsI* by an activated FtsW mutant. Additional *ftsI* alleles were tested to see if they were rescued by *ftsW^M269I^* expression. Some alleles were tested in Fig. 6A, and the others are tested here. Note that among the alleles tested here, only *ftsI^S61F^* is rescued. The bottom row of panels is also presented in Fig. 6A and was included here for comparison. MCI23 (*ftsI23^ts^ recA*::*aadA*) was transformed with compatible plasmids expressing an activated allele of *ftsW* (pSEB429 [P*_204_*::*ftsW^M269I^*]) and the various *ftsI* alleles under arabinose promoter control (derivatives of pBAD33-*ftsI*). Transformants were spot tested on plates at 42°C (to inactivate *ftsI23*^Ts^), and arabinose was added to induce the *ftsI* alleles and increasing concentrations of IPTG to induce *ftsW^M269I^*. Download FIG S8, TIF file, 10.6 MB. Copyright © 2020 Park et al. 2020 Park et al. This content is distributed under the terms of the Creative Commons Attribution 4.0 International license.

### Interaction between FtsL and FtsWI.

**FIG 7:**
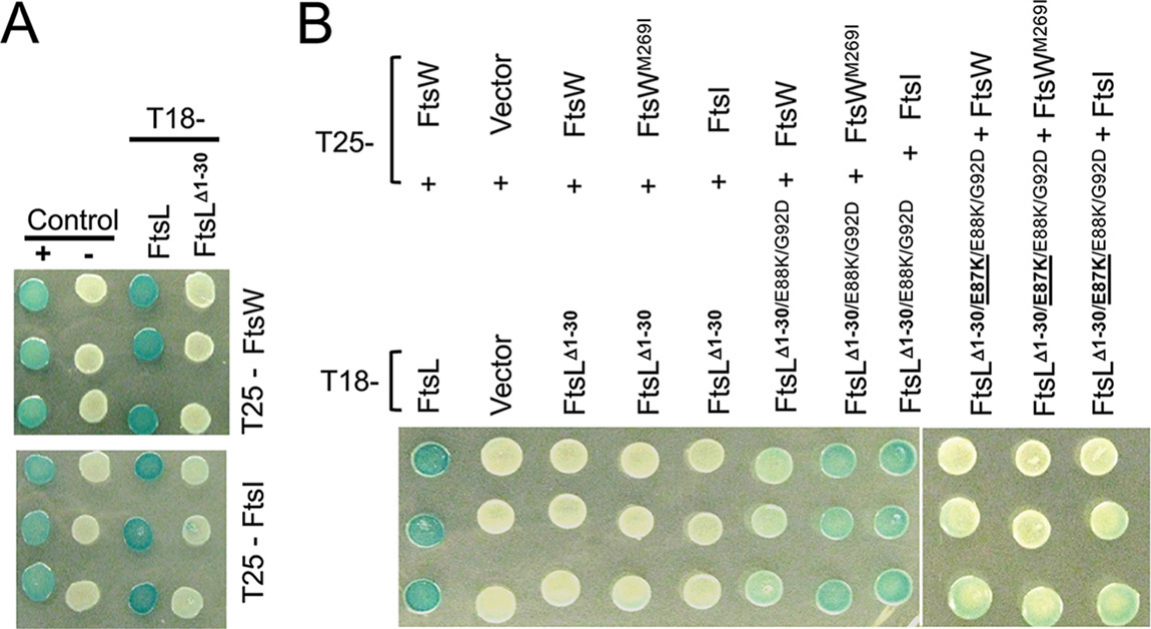
Interaction between FtsL and FtsWI assessed with the BACTH system. (A) Effect of ^cyto^FtsL on the interaction between FtsL and FtsWI. Strain DHM1 was transformed with plasmids carrying various alleles of *ftsL* (pUT18C derivatives) and plasmids expressing *ftsW* or *ftsI* (pKT25 derivatives). Three transformants were picked and spot tested for each pair of constructs. The positive control contained plasmids pUT18C-zip and pKT25-zip, whereas the negative control contained the corresponding empty vectors. (B) The effect of activation and dominant negative mutations in *ftsL* on the interaction of FtsL*^Δ^*^1-30^ or FtsL^L24K/I28K^ with FtsW and FtsI. Three transformants from each transformation of DHM1 with plasmids carrying the f*tsL* and *ftsW* or *ftsI* alleles were spotted on plates containing the color indicator. The *ftsL* alleles were contained in pUT18C, and the *ftsW* and *ftsI* alleles were in pKT25. The plates were photographed after overnight incubation.

Our results point to an interaction between the cytoplasmic domain of FtsL and FtsW required for recruitment of FtsWI and between the periplasmic domain of FtsL with FtsI, which is required for activation of FtsWI. To obtain additional support for interactions between the various proteins, we tested the effect of these mutations using the bacterial two-hybrid (BACTH) system. We observed strong interactions between FtsL and FtsW and between FtsL and FtsI, which were eliminated when the cytoplasmic domain of FtsL was deleted, consistent with ^cyto^FtsL being required for recruiting FtsWI (FtsL^Δ1-30^; Fig. 7A). Elimination of these interactions allowed us to use FtsL^Δ1-30^ to assess the effects of the activation mutations in *ftsL* and *ftsW* on the interactions. Although the *ftsW* activation mutation had little effect, the addition of two *ftsL* activation mutations resulted in a strong interaction between FtsL^Δ1-30^ and FtsI and a weaker interaction between FtsL^Δ1-30^ and FtsW (Fig. 7B). The strong interaction with FtsI suggests it interacts with FtsL, where the weak interaction with FtsW suggests that FtsW is an intermediate. Importantly, the further addition of a dominant negative mutation (*ftsL^E87K^*) eliminated the interaction conferred by the activation mutations. This FtsL variant with three amino acid substitutions was stable, as it interacted with FtsQ as well as the WT FtsL (Fig. S6F). These effects with FtsL^Δ1-30^ were also observed with FtsL^L24K/I28K^ (Fig. S6G). The effects of these *ftsL* mutations in the BACTH system correlate with the effects these mutations have on the rescue of FtsL^Δ1-30^ and FtsL^L24K/I28K^; the *ftsL* activation mutations promote rescue which is negated by an *ftsL* dominant negative mutation (Fig. 4A and Fig. S6D, respectively).

### Rescue of Δ*ftsL* by MalF-FtsL and FtsW-FtsK fusions.

Next, we tested if the periplasmic portion of FtsL transported to the periplasm could activate FtsWI in the absence of full-length FtsL. To do this, a MalF-FtsL fusion was constructed under the control of an IPTG-inducible promoter in which the cytoplasmic and transmembrane (TM) domains of FtsL were replaced with the corresponding regions of MalF (^cyto/TM^MalF-^peri^FtsL). In contrast to FtsL^Δ1-30^, this MalF-FtsL fusion was not dominant negative (Fig. S9A), indicating that the TM region of FtsL must be present for the fusion to displace FtsL from the FtsQLB complex and disrupt FtsW recruitment. This is consistent with the TM region of FtsL being unique (27) and the TMs of FtsL and FtsB being required for these proteins to interact (16, 18). Furthermore, the MalF-FtsL fusion was unable to complement an *ftsL* depletion strain even if the strain carried an *ftsW^M269I^* mutation and the *ftsL* construct carried the two activation mutations (Fig. S9B). This was expected since FtsW would not be recruited.

10.1128/mBio.03012-20.9 FIG S9 Characterization of FtsL fusions. (A) The MalF-FtsL fusion lacks toxicity and fails to complement an *ftsL* depletion strain. The left panel tests for complementation, and the right panel tests for toxicity. SD439 (*ftsL*::*kan*/pSD296 [P*_ara_*::*ftsL*]) containing pKTP100 (P*_tac_*::*ftsL*) derivatives expressing various alleles of *ftsL* were incubated without arabinose or IPTG (basal expression from pKTP100 [P*_tac_*::*ftsL*] is sufficient for complementation) to test for complementation. In the right panel, both arabinose and IPTG were added. Arabinose induces WT *ftsL* from pSD296 (P*_ara_*::*ftsL*), whereas IPTG induces the *ftsL* allele from pKTP100 (P*_tac_*::*ftsL*). The *ftsL^L24K/I28K^* allele was added as a control to demonstrate toxicity. The lack of toxicity indicates that the MalF-FtsL fusion fails to form a complex with FtsQB. (B) FtsL fusions are unable to complement an *ftsL*-depleted strain. Fusion of the periplasmic domain of FtsL to the MalF cytoplasmic and transmembrane domains (with or without *ftsL* activation mutations) was tested for the ability to complement an *ftsL* depletion strain. To do this, PK247-4 (*ftsW^M269I^ ftsL*::*kan*/pSD296 [P*_ar_*_a_::*ftsL*]) containing derivatives of pKTP100 (P*_tac_*::*ftsL*) carrying various fusions in place of *ftsL* was spot tested on plates with IPTG. Download FIG S9, TIF file, 10.6 MB. Copyright © 2020 Park et al. 2020 Park et al. This content is distributed under the terms of the Creative Commons Attribution 4.0 International license.

**FIG 8:**
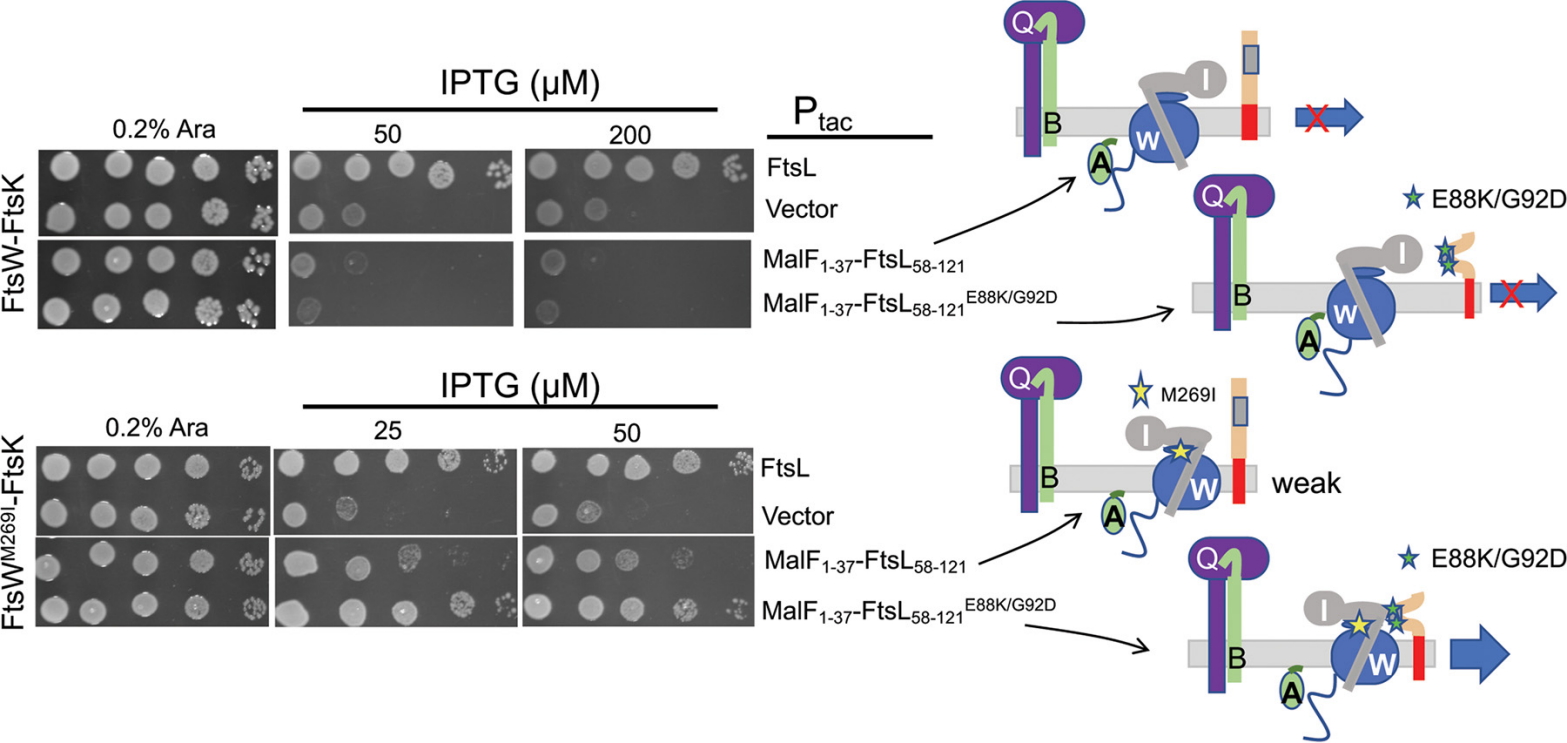
Activation mutations allow a *malF-ftsL* fusion to complement Δ*ftsL* in the presence of a *ftsW-^cyto^ftsK* fusion. Plasmid pKTP103 (P*_tac_*::*malF^1-37^-ftsL^58-121^-6xhis*) was introduced into an *ftsL* depletion strain (SD439 *ftsL*::*kan*/pSD296 [P*_ara_*::*ftsL*]) in the presence of a plasmid constitutively expressing a FtsW-FtsK^cyto^ fusion without or with an activation mutation (pND16 [P*_ftsK_*::*ftsW*-*^cyto^ftsK*] or pND16* [P*_ftsK_*::*ftsW^M269I^*-*^cyto^ftsK*], respectively). The strains were spot tested on plates without arabinose (to deplete WT *ftsL*) and in the presence of IPTG (to induce *malF-ftsL*) (with or without the activation mutations [*ftsL^E88K^* and *ftsL^G92D^*]). The cartoon to the right depicts the activity of the FtsL constructs.

Since the MalF-FtsL fusion cannot cooperate with FtsQB to recruit FtsW, we used an FtsW-^cyto^FtsK fusion which complements an *ftsK* deletion mutant, as well as a *ftsW* deletion mutant, indicating it is targeted directly to the Z ring and bypasses FtsQLB for recruitment (28 and data not shown). This MalF-FtsL fusion was unable to rescue the growth of a strain depleted for FtsL and containing FtsW-^cyto^FtsK, even if the fusion carried both *ftsL* mutations (Fig. 8, top panel). The inability to activate the FtsW-^cyto^FtsK fusion could be for a variety of reasons, including that FtsB is uncoupled from FtsL, and the FtsW-^cyto^FtsK likely competes with endogenous FtsW for FtsI. Nonetheless, the MalF-FtsL fusion with the two activation mutations was able to rescue an FtsL-depleted strain containing the FtsW-^cyto^FtsK fusion with the *ftsW^M269I^* mutation. (Fig. 8). Even the MalF-FtsL fusion without the *ftsL* activation mutations partially rescued growth at higher induction levels. These results suggest that MalF-FtsL acts on FtsI associated with the FtsW^M269I-cyto^FtsK fusion that is already at the Z ring to rescue growth. Since the activation mutations in *ftsL* potentiate MalF-FtsL activity, it suggests that in addition to making AWI available within the FtsQLB complex, they may also alter the structure of AWI to enhance its interaction with FtsWI.

## DISCUSSION

Here, we investigated how septal PG synthesis in the divisome is activated by FtsN and identified a critical and unique role for FtsL. Our results are consistent with the recruitment of FtsW requiring the cytoplasmic domain of FtsL and the activation of FtsWI being dependent upon AWI in the periplasmic domain of FtsL. Based upon the seminal work by the de Boer lab, which is supported by the work from the Bernhardt lab (10, 11) and our results (12) and those here, we propose that the arrival of FtsN leads to a conformational change in the FtsQLB complex that makes the AWI domain of FtsL, as defined by the dominant negative *ftsL* mutations, available to activate FtsWI by acting through FtsI. Furthermore, activation mutations in the CCD domain of FtsL as well as those in FtsB mimic FtsN action to cause a conformational change in FtsQLB to expose the AWI domain. This model is supported by the ability of activation mutations in *ftsL* to rescue FtsL mutations (FtsL^Δ1-30^ and FtsL^L24K/I28K^) deficient in FtsWI recruitment and by the dominant negative mutations in *ftsL* (*ftsL^L86F/E87K^*) negating the rescue. The effects of these *ftsL* mutations (both activation and dominant negative) on the rescue of the FtsL mutants correlates with their effects on the observed interaction between FtsL and FtsWI in the BACTH system. The model is also supported by the ability of the expression of *ftsI* to rescue FtsL^Δ1-30^ more efficiently than *ftsW*. Furthermore, FtsL acting on FtsI to activate FtsW is supported by the ability of an active FtsL mutant to rescue FtsI mutants not rescued by an activated FtsW. Thus, we propose that as a result of ^E^FtsN action, the AWI domain of FtsL becomes available to interact with FtsI within the FtsWI complex to activate FtsW and synergizes with ^cyto^FtsL in stabilizing the FtsWI complex in the divisome. Thus, FtsL within the FtsQLB complex functions as a clamp to maintain FtsWI in the divisome.

### The AWI domain.

Altering seven residues in the periplasmic domain of FtsL produced a dominant negative phenotype. All, except for one, are clustered together around the CCD. We focused on L86 and E87 and believe these are central to the AWI domain. This suggestion is based upon the following: (i) L86 and E87 are relatively well conserved, and loss of the negative charge at E87 is sufficient to produce a dominant negative allele (suggesting disruption of an interaction); (ii) the *ftsL^L86F^* or *ftsL^E87K^* dominant negative mutations are not suppressed by activation mutations (*ftsL^E88K^* or *ftsB^E56A^*) or *ftsN* overexpression; (iii) ^cyto^FtsL mutants that fail to recruit FtsWI are rescued by the addition of two *ftsL* activation mutations (*ftsL^E88K/G92D^*); (iv) the rescue of ^cyto^FtsL mutants by *ftsL* activation mutations or overexpression of *ftsW^M269I^* is negated by adding dominant negative mutations (*ftsL^L86F^* or *ftsL^E87K^*); and (v) the effects of these mutations on the interaction of FtsL with FtsWI in the BACTH system correlate well with the effects of these mutations on the rescue of FtsL^Δ1-30^. It is likely that other regions of FtsL (and FtsB), such as the transmembrane domains (TM) and coiled coil domains, are also involved in interaction with FtsWI.

The dominant negative mutations in *ftsL* are less responsive to FtsN, and most overlap the CCD domain, which was defined by hyperactive mutations that are less dependent upon FtsN (10, 11). Despite the overlap, the residues comprising each domain mostly lie on opposite sides of a putative helix (Fig. 2C). The dominant negative mutations appear to be unique to *ftsL*, as we were unable to isolate any such mutations in *ftsB*. Although previous studies suggested that FtsN induces a change in FtsQLB from an OFF to ON conformation (10), it was not clear how this switch led to activation of FtsWI. Here, we identify the AWI domain of FtsL and suggest that the function of the conformational switch is to make AWI available to interact with FtsWI. Since FtsQLB may be a dimer, the conformational change could involve disruption of this dimer which makes AWI available; however, this will require further study (15, 16, 29).

Additional evidence for the unique importance of the periplasmic domain of FtsL comes from the ability of the MalF-^peri^FtsL fusion to rescue an FtsW-^cyto^FtsK fusion when both are carrying activation mutations. The FtsW-^cyto^FtsK fusion is unable to support growth in the absence of FtsL even though it localizes. On the other hand, the MalF-^peri^FtsL fusion does not form a complex with FtsQB, so it is not recruited to the divisome. Nonetheless, the ability of the MalF-^peri^FtsL to collaborate with FtsW-^cyto^FtsK (when both are carrying activation mutations) to rescue growth suggests that the periplasmic domain of FtsL is able to act on FtsW-^cyto^FtsK complexed with FtsI.

While this paper was under review, Marmont and Bernhardt (30) reported that FtsLB was sufficient to activate PG synthesis by FtsWI *in vitro*, providing biochemical evidence for an activation model. They also isolated dominant negative mutations in *ftsL* which overlap those we isolated, even though their work was done in Pseudomonas aeruginosa and FtsL is not so highly conserved at the sequence level. Some, but not all, of the dominant negative mutants prevented activation *in vitro*. However, the *in vitro* system does not fully recapitulate the *in vivo* regulation, as FtsN was not required for activation.

### Conditions that rescue FtsL^Δ1-30^ favor interaction between the AWI domain of FtsL and FtsI.

Surprisingly, loss of the cytoplasmic domain of FtsL, which prevents recruitment of FtsWI and blocks cell division, could be rescued by activation mutations in the periplasmic domain of FtsL as well as by overexpression of FtsN. We reasoned that these activation conditions expose an interaction that normally occurs when the divisome is activated and that this interaction is able to compensate for the loss of ^cyto^FtsL to recruit FtsWI. In support of this model, *ftsL* activation mutations in *ftsL*^Δ1-30^ promoted interaction between FtsL and both FtsW and FtsI. Also, these interactions were negated by the addition of a dominant negative mutation. These results suggest that FtsL within the FtsQLB complex functions as a transmembrane clamp (Fig. 1) to stabilize the active FtsWI complex within the divisome. The cytoplasmic domain of FtsL is required to recruit FtsW, which in turn recruits FtsI. FtsN action then frees the AWI domain to interact with FtsI and, as we have shown here, this domain, when freed, is able to rescue *ftsL*^Δ1-30^, indicating FtsWI recruitment is restored.

Since it is likely FtsQLB exists in equilibrium between ON and OFF states, we reasoned that expression of the downstream partner might also rescue *ftsL*^Δ1-30^ by capturing the ON form and pulling the equilibrium in that direction. In fact, the active form of FtsW was effective in rescuing *ftsL*^Δ1-30^, much more so than FtsW. However, expression of FtsI was very effective in rescuing *ftsL*^Δ1-30^ and much more so than overexpression of FtsW, which barely rescued at high overexpression. This (i) suggested that FtsI is the direct downstream target of AWI, (ii) suggested that rescue by expression of FtsW likely involves formation of an FtsWI complex recruited by AWI, and (iii) raises the possibility that the activated form of FtsW interacts more strongly with FtsI. Consistent with the rescue of *ftsL*^Δ1-30^ by expression of FtsI or activated FtsW being dependent upon the interaction of AWI with FtsWI in the periplasm, it was prevented by the addition of the dominant negative *ftsL* mutations. This is in stark contrast to the suppression of the dominant negative mutations in full-length *ftsL* by activated FtsW. When full-length FtsL is present, an FtsW activated by mutation is recruited normally and no longer requires the activation signal so the dominant negative mutations do not prevent the rescue (although rescue is aided by overexpression of the activated FtsW). On the other hand, FtsW and FtsI are unable to rescue, as they still depend upon the AWI signal.

Our results suggest that FtsWI forms a dynamic complex, and it is this complex that is preferred by FtsL. If FtsWI formed a stable complex, then overexpression of FtsW would be toxic, as excess FtsW would titrate FtsI away from the division site inhibiting division. However, overexpression of *ftsW* is not toxic in WT cells and it only weakly rescued *ftsL*^Δ1-30^. Also, when FtsQLB is overexpressed and purified, FtsW and FtsI only copurify efficiently if they are both expressed, indicating that the FtsWI complex interacts more stably with FtsQLB than FtsW or FtsI alone (31). Thus, overexpression of FtsW may favor complex formation with FtsI and septal localization to rescue *ftsL*^Δ1-30^. More efficient rescue by an activated FtsW could be due to it favoring complex formation with FtsI. On the other hand, the rescue of *ftsL*^Δ1-30^ by FtsI expression is probably due to a direct interaction with AWI; otherwise, the rescue of *ftsL*^Δ1-30^ by FtsW and FtsI should be comparable, since overexpression of either should promote complex formation.

The product of FtsN action is an activated FtsWI complex in which both FtsW and FtsI are active. The ability of active FtsW mutants to suppress the dominant negative FtsL mutants (and bypass the periplasmic signal) indicates that an active FtsW leads to an active FtsWI complex. Among previously isolated FtsI mutants, we found some that were rescued by both an active FtsW mutant and an active FtsL mutant. However, an activated FtsL rescued two additional FtsI mutants that could not be rescued by an activated FtsW. This suggests that AWI acts on FtsI to activate FtsW and does not act directly on FtsW. In other words, the signal transmission from FtsN is from ^peri^FtsL → FtsI → FtsW and not ^peri^FtsL → FtsW → FtsI.

Although *in vitro* results suggest that FtsQLB acts as an inhibitor with FtsL inhibiting PBP1b and FtsQ inhibiting FtsI and therefore FtsW (31), our results are more compatible with a model in which AWI is sequestered within FtsQLB and becomes available upon FtsN action to activate FtsWI. The findings that *ftsL* activation mutations rescue FtsL^Δ1-30^ and promote interaction between FtsL^Δ1-30^ and FtsWI in the BACTH are consistent with the FtsL-FtsWI interaction activating FtsWI. This conclusion is also supported by the *ftsL* dominant negative mutations negating both of these activities.

### Comparison of models for divisome and elongasome activation.

It is interesting to compare our model for FtsWI activation with the model proposed for activation of the RodA-PBP2 pair that are part of the elongasome (homologous to FtsW-FtsI [PBP3]). That model is based upon (i) the structure of the MreC-PBP2 complex (32) and (ii) the finding that mutations that bypass *mreC* and activate RodA-PBP2 map to the nonpenicillin (nPD) or pedestal domain of PBP2 (33). It is thought that these mutations mimic the binding of MreC to PBP2, altering the conformation of PBP2, which results in the activation of RodA. In this way, the activity of RodA and PBP2 are coupled to ensure RodA only makes glycan strands when its cognate PBP is present. This is remarkably similar to our model for FtsW-FtsI (PBP3) activation with FtsL (with possibly a supporting role for FtsB) being analogous to MreC. The isolation of FtsW activation mutants that bypass FtsN suggests that an activated FtsW results in an active FtsI. Furthermore, an active FtsW mutant can rescue dominant negative FtsL mutants (i.e., bypass the signal from FtsN), indicating FtsI is also activated. Thus, we propose that FtsN action alters the conformation of FtsQLB so that AWI becomes available to interact with FtsI, leading to conformational change in FtsI that activates FtsWI’s enzymatic activities.

## MATERIALS AND METHODS

### Bacterial strains and growth conditions.

Bacterial strains are listed in Table S1A. JS238 [*MC1061*, *araD* Δ(*ara leu*) *galU galK hsdS rpsL* Δ(*lacIOPZYA*)*X74 malP*::*lacI^Q^ srlC*::Tn*10 recA1*] was primarily used for screening for *ftsL* and *ftsB* dominant negative mutations and as a host for most cloning experiments. W3110 was used to generate SD399, SD439, and SD285. To construct SD399 [*W3110*, *ftsL*::*kan/*pSD256], P1 phage grown on BL156 [*ftsL*::*kan*/pJH2] was used to transduce *ftsL*::*kan* into W3110/pSD256 by selecting for Kan resistance on LB agar plates containing 25 μg/ml kanamycin, 50 μg/ml spectinomycin, and 8 mM sodium citrate at 30°C. Several colonies were subcloned onto fresh plates of the same composition at 30°C and were further screened for temperature sensitivity at 42°C. SD439 was created by transforming SD399 with pSD296 (P*_ara_*::*ftsL*) and selecting the transformants that grow at 42°C (to remove pSD256) in the presence of 10 μg/ml chloramphenicol and 0.2% arabinose. Colonies were streaked and further tested for spectinomycin sensitivity (indicating loss of pSD256). Construction of SD285 [*leu*::Tn*10 bla lacI*^q^ *P*_207_‐*gfp‐ftsI*] involved transduction with P1 phage grown on EC436 [MC4100 Δ(λ*attL‐lom*)::*bla lacI*^q^ *P*_207_‐*gfp‐ftsI*] into S3 (W3110 *leu*::Tn*10*). Transductants were selected on LB agar plates containing 25 μg/ml ampicillin and 10 μg/ml tetracycline. Expression of GFP-FtsI was confirmed in the transductant clones by induction with 10 to 20 μM IPTG. SD247 (W3110 *ftsW^M269I^*) was previously described (12), and PK247-4 [SD247 *ftsL*::*kan/*pSD296] was generated by P1 transduction of *ftsL*::*kan* from the SD399 donor to the recipient strain SD247/pSD296 [P_ara_::*ftsL*] and by selecting Kan resistance and screening for arabinose dependency. PK4-1 (*ftsL*::*kan/*pKTP108 [P_ara_::*ftsL*]) was generated by using the same procedure described above. Unless stated otherwise, Luria-Bertani broth (LB) medium containing 0.5% NaCl was used at the indicated temperatures. For selection on LB agar and growth in LB broth, the following antibiotics and reagents were added at the indicated final concentrations as necessary: ampicillin, 100 μg/ml; spectinomycin, 50 μg/ml; kanamycin, 25 μg/ml; chloramphenicol, 10 μg/ml; tetracycline, 10 μg/ml; IPTG, 10 to 200 μM; glucose, 0.2%; and arabinose, 0.2%.

10.1128/mBio.03012-20.10 TABLE S1 Strains and plasmids used in this study. Download Table S1, DOCX file, 0.04 MB. Copyright © 2020 Park et al. 2020 Park et al. This content is distributed under the terms of the Creative Commons Attribution 4.0 International license.

### Plasmids.

The plasmids are listed in Table S1B. Genomic DNA extracted from the W3110 strain was used as a template to obtain PCR fragments to generate expression plasmids for *ftsL*. To construct the plasmids pKTP100 (P*_tac_*::*ftsL*) and pKTP103 [P*_tac_*::*malF^1-37^ ftsL^58-121^-6xhis*], the *ftsL* open reading frame (ORF) was PCR amplified incorporating a strong ribosome binding site in the forward primers targeting *ftsL*, which included sequences for *ftsL* and *malF*^1-37^, respectively. The PCR fragments were digested with EcoRI and HindIII and ligated into the same sites in pJF118EH. Construction of pKTP104 (*P_T5_*::*ftsL*) and pKTP105 (P_T5_::*ftsL^30-121^*) involved PCR amplification of the *ftsL* ORF, digestion with BamHI and HindIII, and ligation into the same sites in the pQE80L vector (Qiagen). The construction of pKTP108 [*repA*^ts^ P_syn135_::*ftsL*] employed a similar approach to that used for pSD256 (12) except that a strong ribosome binding site was added and the XbaI site was used instead of EcoRI. To create pKTP109, the *ftsI* ORF was PCR amplified and digested with SacI and HindIII, followed by ligation into pBAD33 using sites with compatible overhangs. To generate plasmid pSD296 (P_ara_::*ftsL*), the *ftsL* ORF and its flanking sequences (250 bp) were PCR-amplified, digested with XbaI and HindIII, and ligated into the same sites in the pBAD33 vector. Plasmids pKTP106 (P_ara_::*ftsL*) and pKTP107 (P_ara_::*ftsL^30-121^*) were created by PCR amplification of *ftsL* and *ftsL^30-121^*, respectively, using the primers that contain the same ribosome binding site as in pKTP100. The two PCR fragments were cut with SacI and HindIII and cloned into sites in pBAD33 with compatible overhangs. To create pKTP101 (P_tac_::*ftsB*), the ORF was PCR amplified and digested with EcoRI and HindIII followed by ligation into pJF118EH cut with the same enzymes. The pND16 [P*_ftsK_*::*ftsW-ftsK^179-1329^*] plasmid constitutively expresses the FtsW-FtsK C-terminal fusion protein, and pBL154 (repA^TS^ P_syn135_::*ftsN*) was previously described (10, 34). The overexpression plasmids for FtsN, FtsI, and FtsW and pSEB417 (P_204_::*ftsN*), pSEB420 (P_204_::*ftsI*), and pSEB429 (P_204_::*ftsW*), respectively, were previously described (21, 34). Note that these genes are expressed from their endogenous ribosome binding sites.

The bacterial two-hybrid (BACTH) vectors, pUT18C (cyaA^T18^ fragment) and pKT25 (cyaA^T25^ fragment), were described previously (35). The pUT18C-ftsL (*cya^T18^-ftsL*) and pUT18C-ftsL^30-121^ (*cya^T18^-ftsL^30-121^*) plasmids were generated by ligating PCR-amplified *ftsL* and *ftsL^30-121^* into pUT18C (*cya^T18^*) digested with BamHI and EcoRI, respectively. Construction of pUT18C-ftsW (*cya^T18^-ftsW*) and pKT25-ftsW (*cya^T25^-ftsW*) involved PCR amplification of E. coli *ftsW* ORF and digestion of the fragments with BamHI and KpnI, followed by ligation into the BACTH vectors digested with the same enzymes. pKT25-ftsI (*cya^T25^-ftsI*) was created by similar procedures, but BamHI and EcoRI were used for digestion of PCR fragment and vector. For construction of pKT25-ftsQ (*cya^T25^-ftsQ*), the *ftsQ* ORF was PCR amplified, digested with XbaI and EcoRI, and ligated into pKT25 cut with the same enzymes. All primers are available on request.

### Random and site-directed mutagenesis.

To obtain the *ftsL* and *ftsB* mutant libraries (with a single missense mutation per ORF) an optimal mutation rate (0.3 to 1 base/kb) for 1 μg of template was adopted as recommended in the GeneMorph II random mutagenesis kit (Agilent Technologies). The PCR products were then digested with EcoRI and HindIII and ligated into the pJF118EH vector using the same restriction enzymes. A ligation pool of pJF118EH-ftsL or pJF118EH-ftsB containing putative mutations was transformed into JS238 by electroporation, and transformants were selected on LB plates containing ampicillin (100 μg/ml) at 37°C. A dominant negative phenotype was screened for by screening sensitivity to IPTG. Specific point mutations in *ftsL*, *ftsL^30-121^*, and *ftsW* were introduced into some plasmids by using the QuikChange site-directed mutagenesis kit according to the manufacturer’s instructions (Agilent Technologies).

### Isolation of an allele of *ftsW* that bypasses *ftsN*.

To generate a library of random *ftsW* mutations, *ftsW* was subjected to random PCR mutagenesis and cloned into plasmid pSEB429 (P_204_::*ftsW*) to replace the WT *ftsW*. The mutagenized library (pSEB429M) was transformed into strain SD399 [*ftsL*::*kan*/pSD256 (repA^ts^::*ftsL*)] harboring plasmid pSD296-E87K (P_ara_::*ftsL^E87K^*), and suppressors of FtsL^E87K^ were selected on LB plates with 0.2% arabinose (to induce *ftsL^E87K^*) and 60 μM IPTG (to induce *ftsW*) at 37°C. Fourteen of the surviving clones were purified, retested, and sequenced. Eleven contained a single mutation (E289G), while 3 contained this mutation plus other mutations. The *ftsW^E289G^* mutation was introduced into S3 (W3110, *leu*::*Tn10*) by recombineering. P1 transduction of *ftsN*::*kan* from strain CH34/pMG20 (*ftsN*::*kan*/P_ara_::*^SS^torA-bfp ftsN^71-105^*) into SD488 (*leu*::*Tn10*, *ftsW^E289G^*) was done using a standard procedure. The Kan^R^ transductants had a slightly longer phenotype than a WT strain.

### Helix modeling of the FtsL periplasmic domain.

A secondary structure of FtsL was generated for illustrative purposes. To do this, a crude model of the putative coiled coil region of FtsL was modeled on the coiled coil structure (tropomyosin, 1IC2). Structures were visualized using PyMOL (Molecular Graphics System version 1.2r3pre; Schrödinger, LLC).

### Bacterial two-hybrid analysis.

The *cya* null strain DHM1 [*F-*, *cya-854*, *recA1*, *endA1*, *gyrA96 (Nal^r^*), *thi1*, *hsdR17*, *spoT1*, *rfbD1*, *glnV44(AS)*] was simultaneously transformed with plasmids pKT25-ftsW or pKT25-ftsI and pUT18C-ftsL (or-ftsL^30-121^), carrying wild-type or mutant *ftsW* and *ftsL* alleles, and grown overnight at 30°C on LB plates containing 0.2% glucose, 25 μg/ml kanamycin, and 100 μg/ml ampicillin. Colonies from the LB plates were diluted in 300 μl volume of LB broth and spotted onto fresh LB plates supplemented with 25 μg/ml kanamycin, 100 μg/ml ampicillin, 40 μg/ml 5-bromo-4-chloro-3-indoyl-β-d-galactopyranoside (X-Gal), and 0.5 mM IPTG. The color changes were recorded after overnight incubation at room temperature at 30°C.

### Microscopy.

The dominant negative effects of the FtsL mutants on cell division were assessed using phase-contrast microscopy by monitoring the degree of filamentation. JS238 containing pKTP100 or derivatives carrying *ftsL* mutations was grown overnight at 30°C in the presence of 100 μg/ml ampicillin and 0.2% glucose. The cultures were diluted 1/200 to 1/500 in fresh LB medium containing 100 μg/ml ampicillin at 30°C. At an optical density at 540 nm (OD_540_) of ∼0.02, 50 μM IPTG was added, and cell morphologies were analyzed 2 h later.

To visualize GFP-FtsI localization, SD285 (*leu*::Tn*10 bla lacI*^q^ *P*_207_‐*gfp‐ftsI*) containing pKTP106 (P_ara_::*ftsL*) or derivatives with the *ftsL^E87K^* or *ftsL^A90E^* mutations was grown overnight at 30°C in LB medium containing 50 μg/ml ampicillin and 10 μg/ml chloramphenicol. The overnight cultures were diluted 1/200 to ∼1/500 in fresh LB medium containing the same antibiotics, 0.2% arabinose, and 10 to 20 μM IPTG and were incubated at 37°C until the OD_540_ was ∼0.4. Cells were immobilized on an LB agarose pad, and the localization of GFP-FtsI was recorded using a cooled charge-coupled-device (CCD) camera and processed using Metamorph (Molecular Devices) and Adobe Photoshop.
