# Supplementary figures and images for: Essential Role for FtsL in Activation of Septal Peptidoglycan Synthesis
Source: mBio. 2020 Dec 8;11(6):e03012-20. doi: 10.1128/mBio.03012-20 (PMC7733951; doi:10.1128/mBio.03012-20)

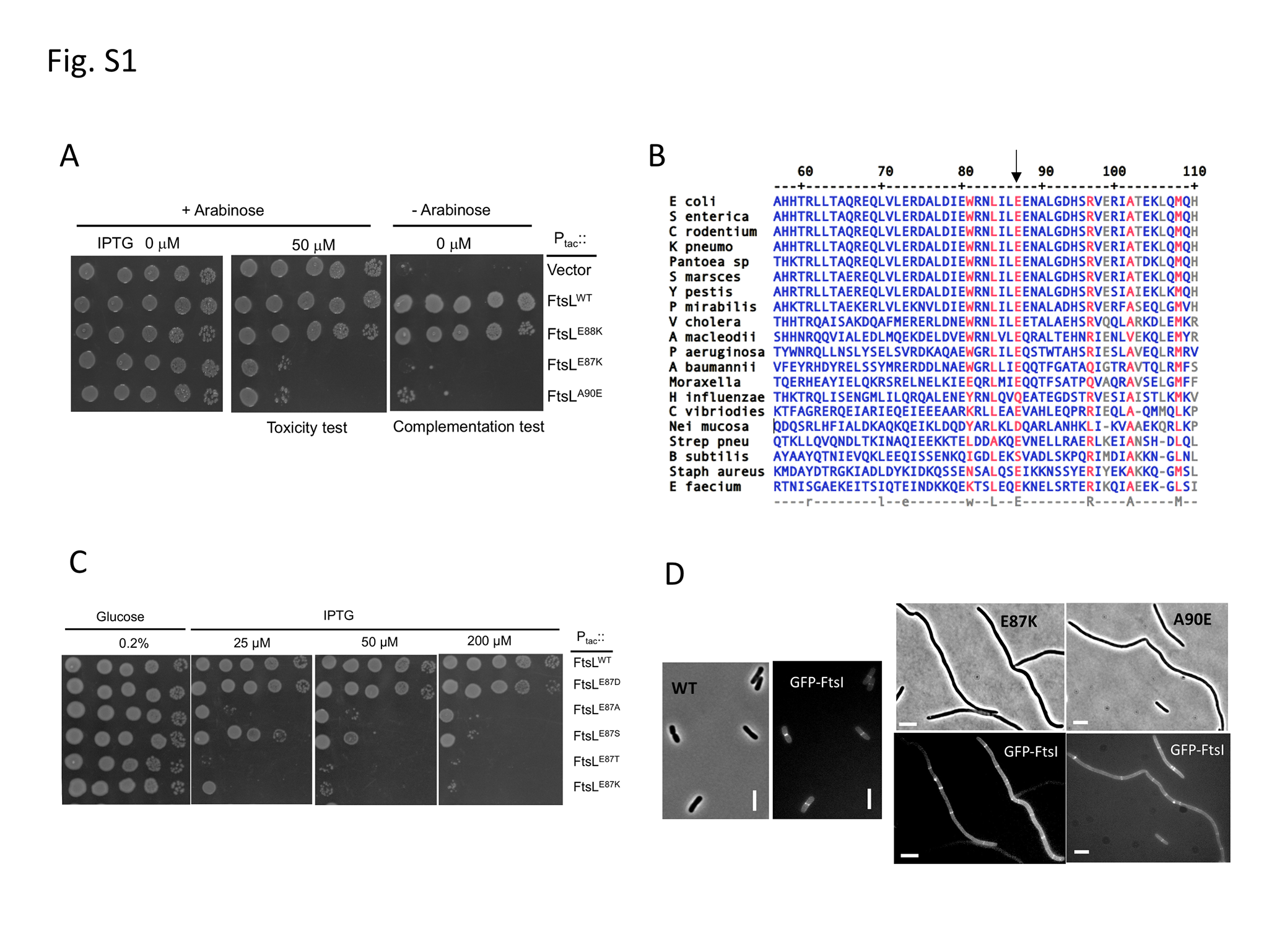

Supplement: FIG S1 [file mBio.03012-20-sf001.tif]

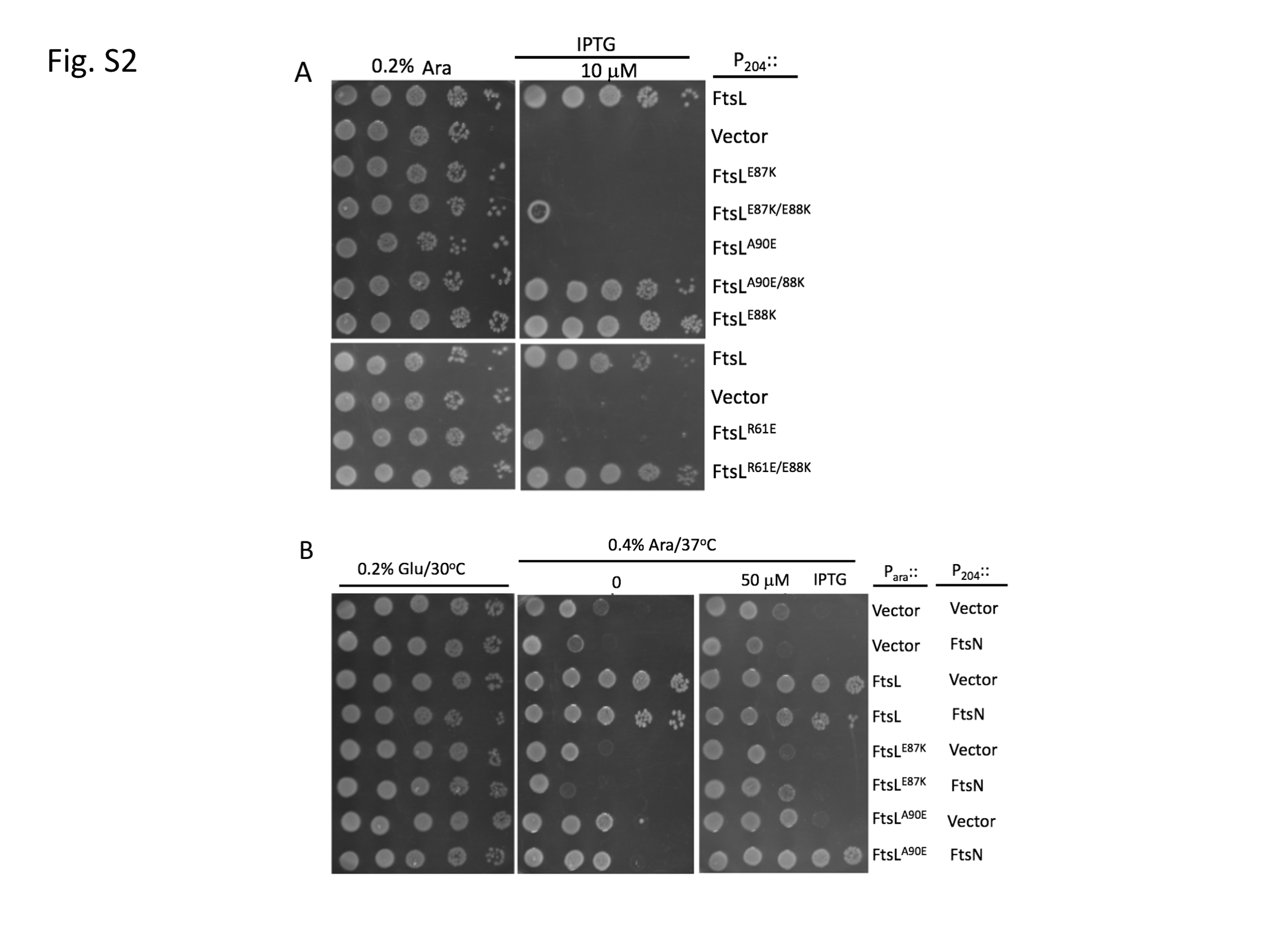

Supplement: FIG S2 [file mBio.03012-20-sf002.tif]

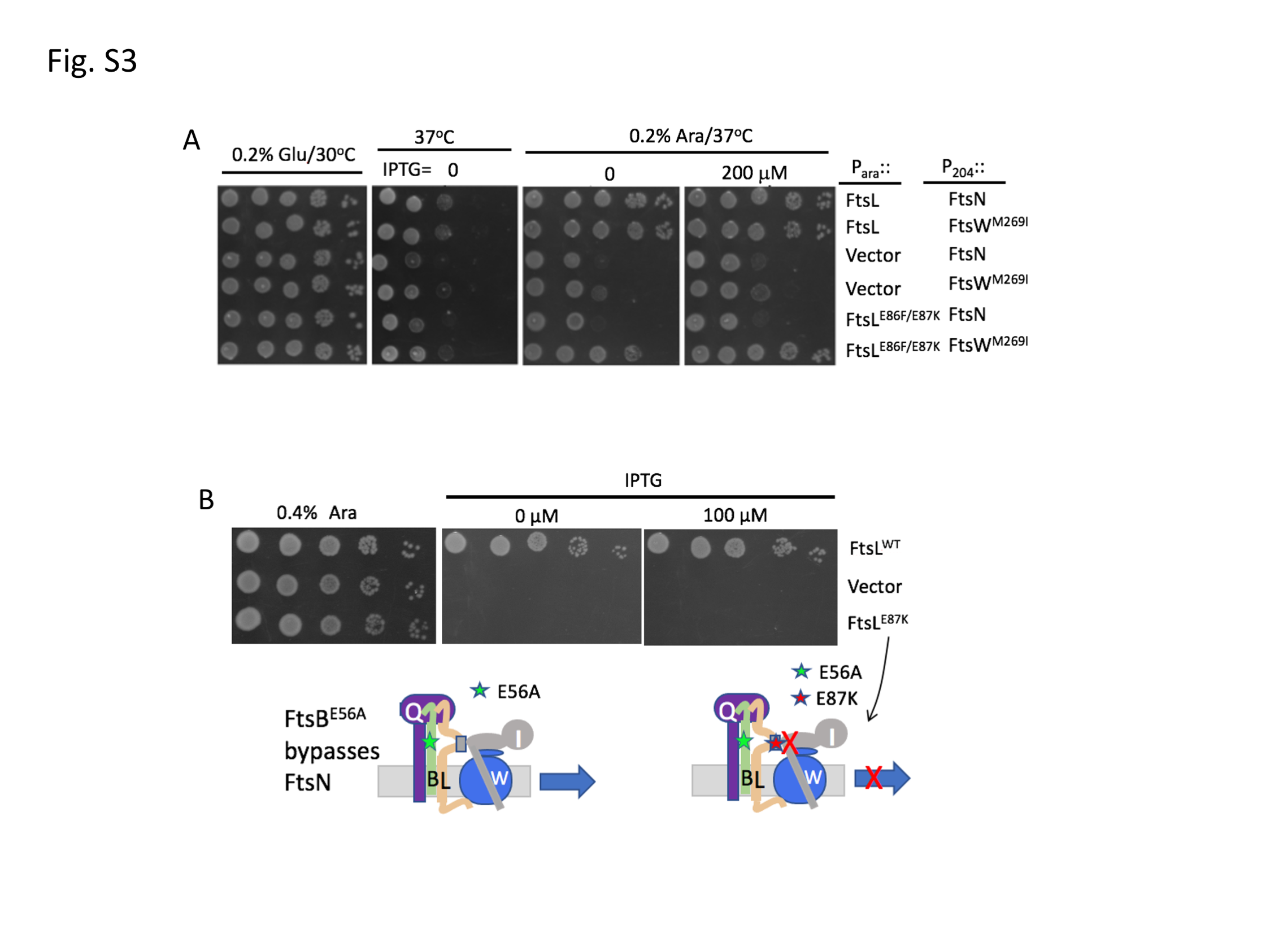

Supplement: FIG S3 [file mBio.03012-20-sf003.tif]

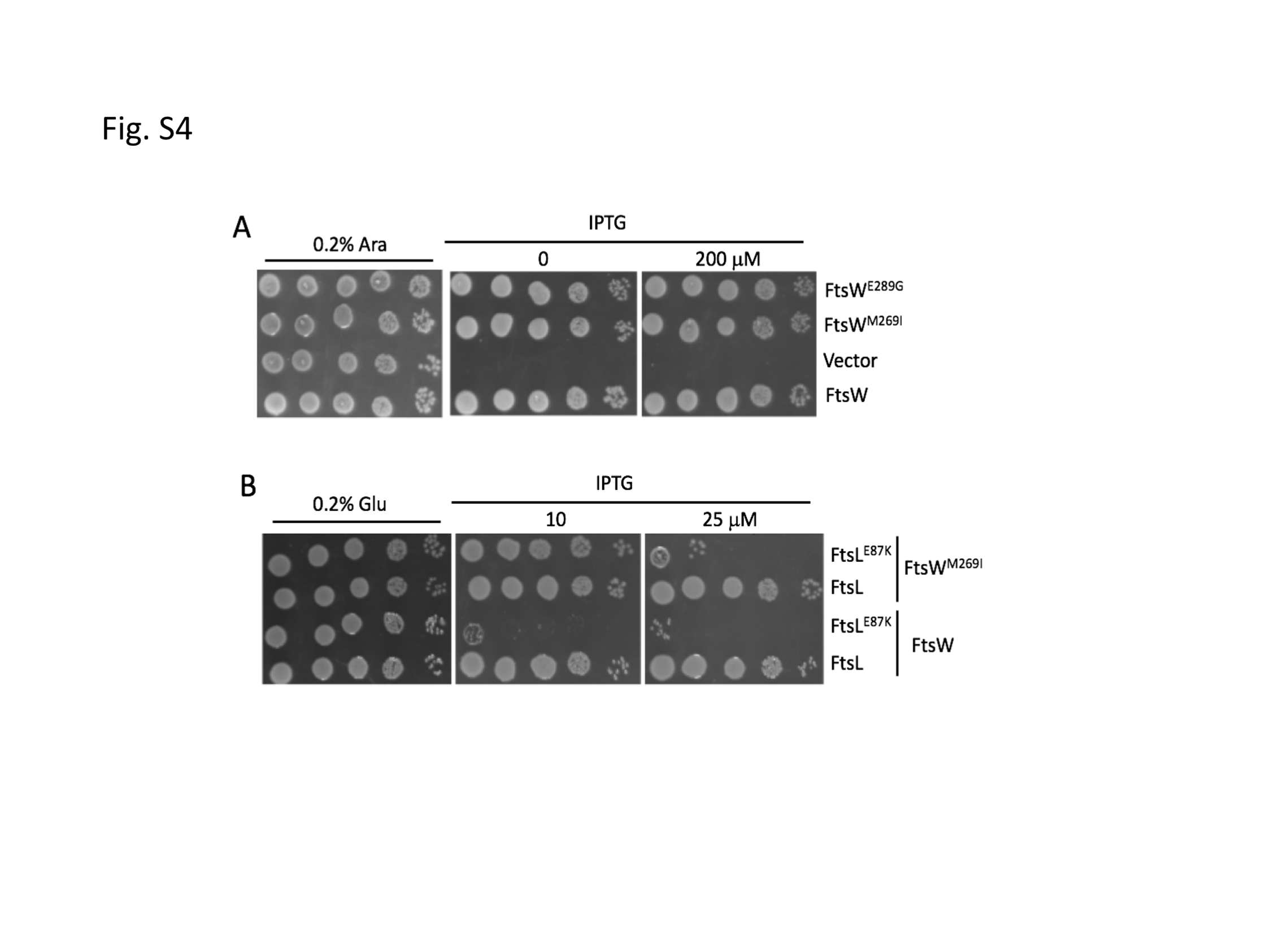

Supplement: FIG S4 [file mBio.03012-20-sf004.tif]

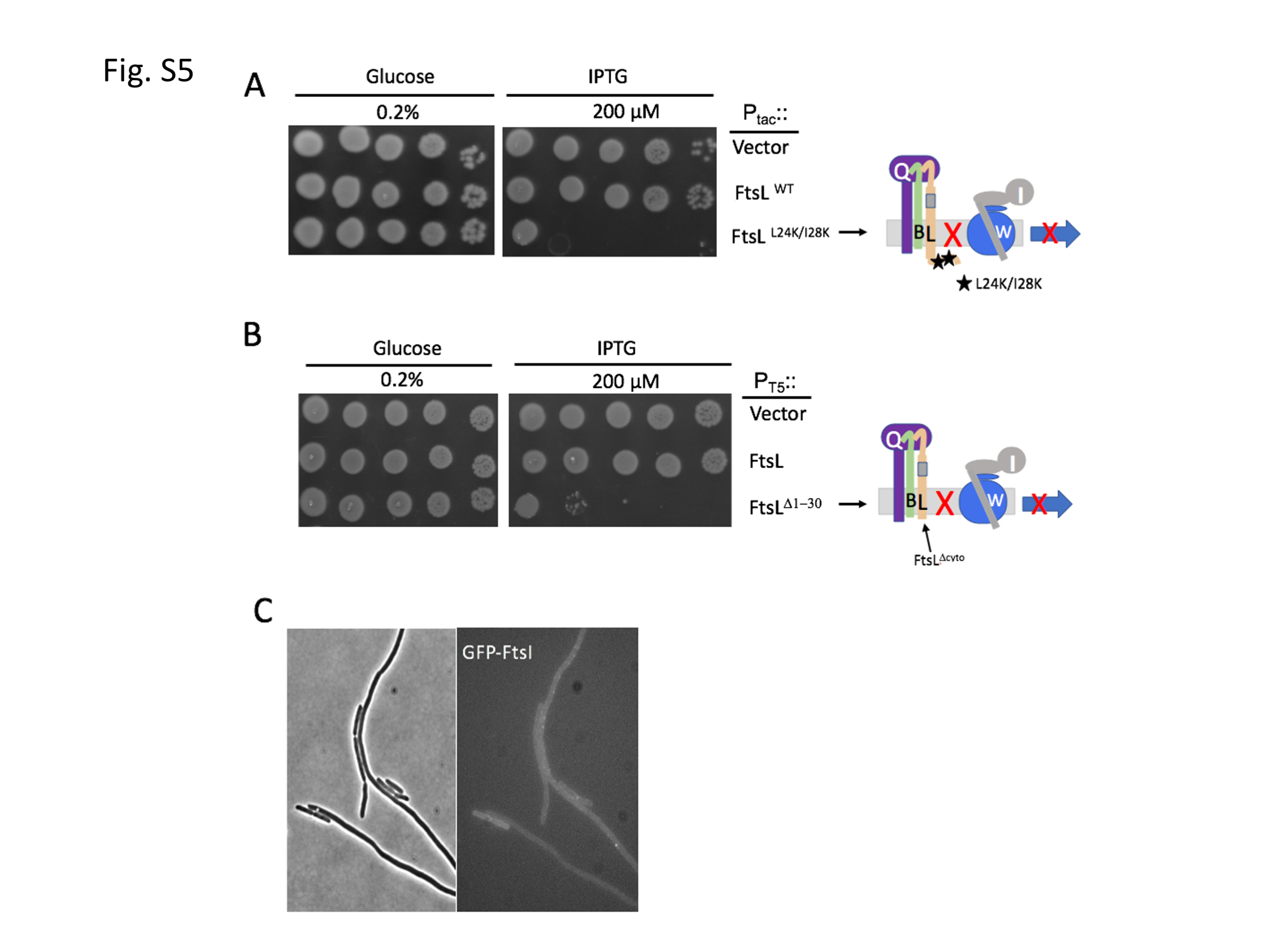

Supplement: FIG S5 [file mBio.03012-20-sf005.tif]

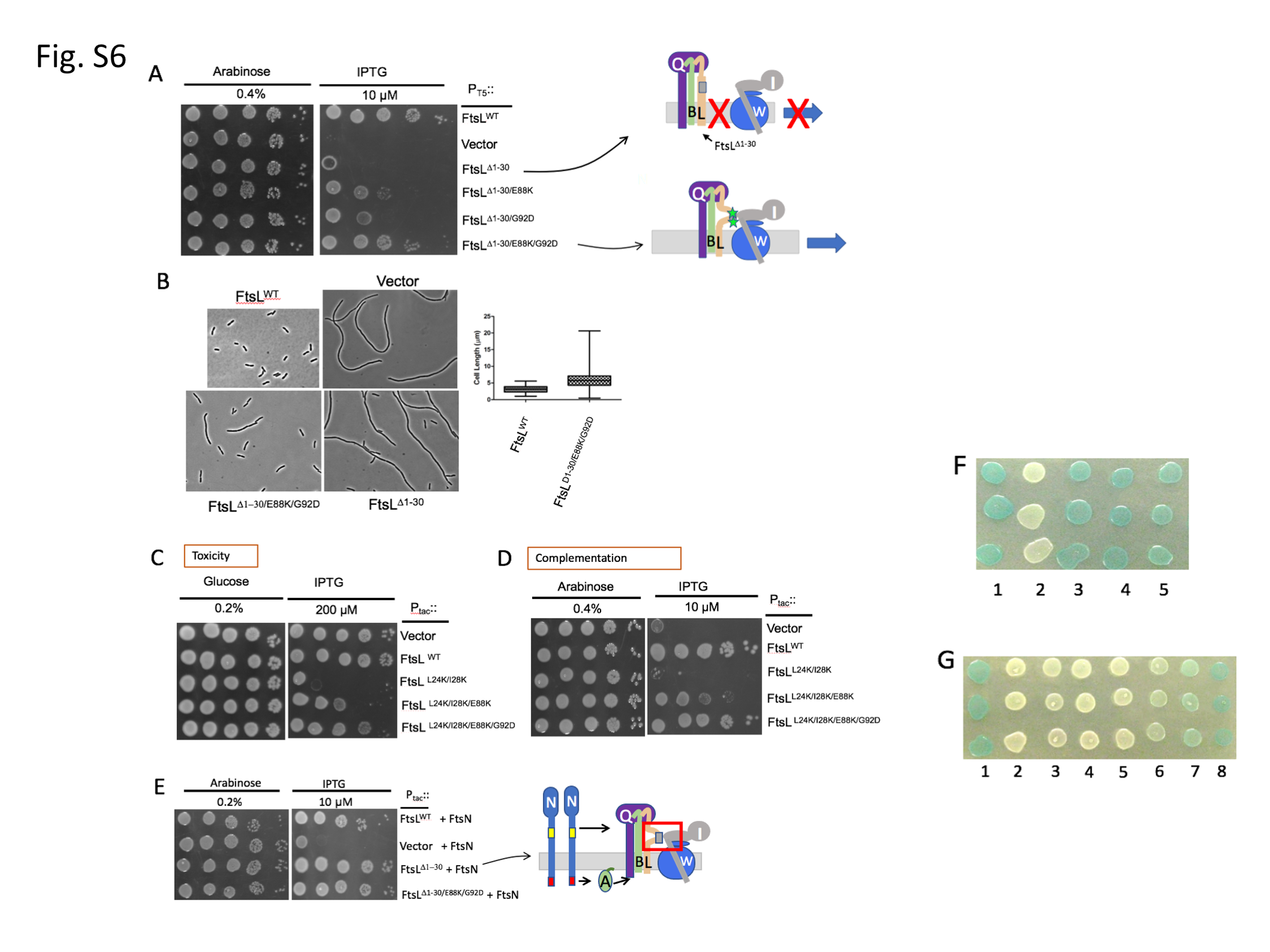

Supplement: FIG S6 [file mBio.03012-20-sf006.tif]

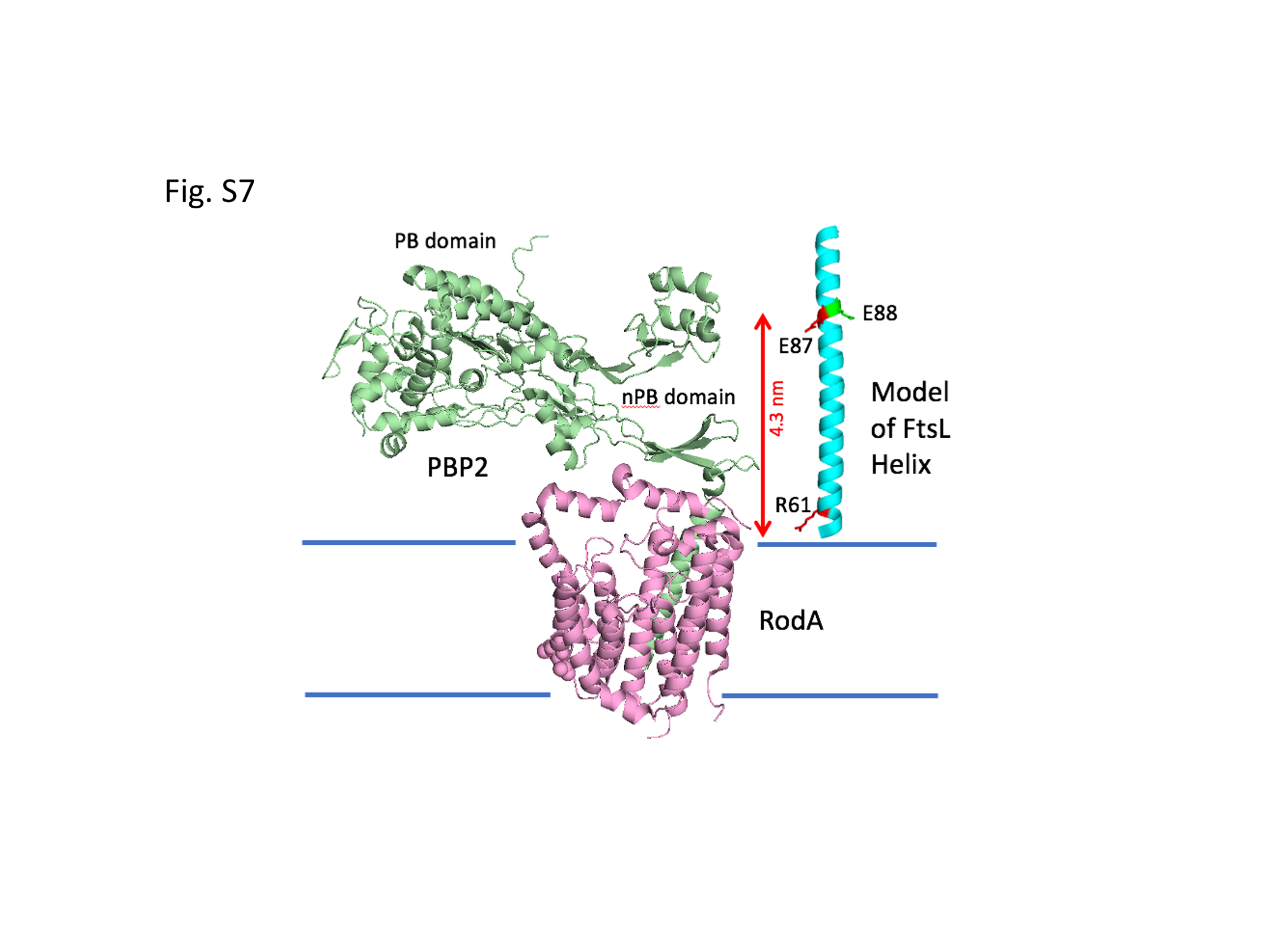

Supplement: FIG S7 [file mBio.03012-20-sf007.tif]

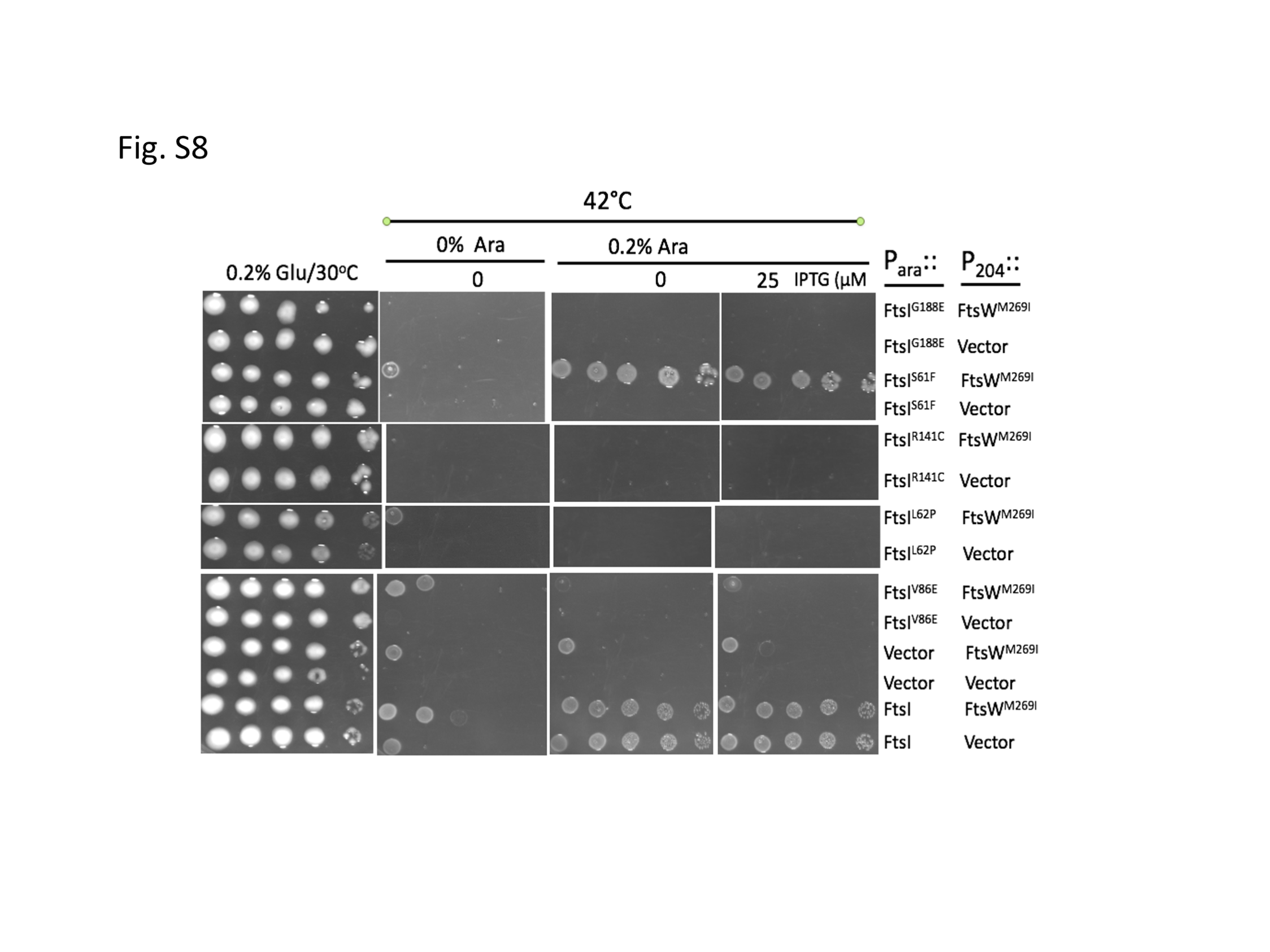

Supplement: FIG S8 [file mBio.03012-20-sf008.tif]

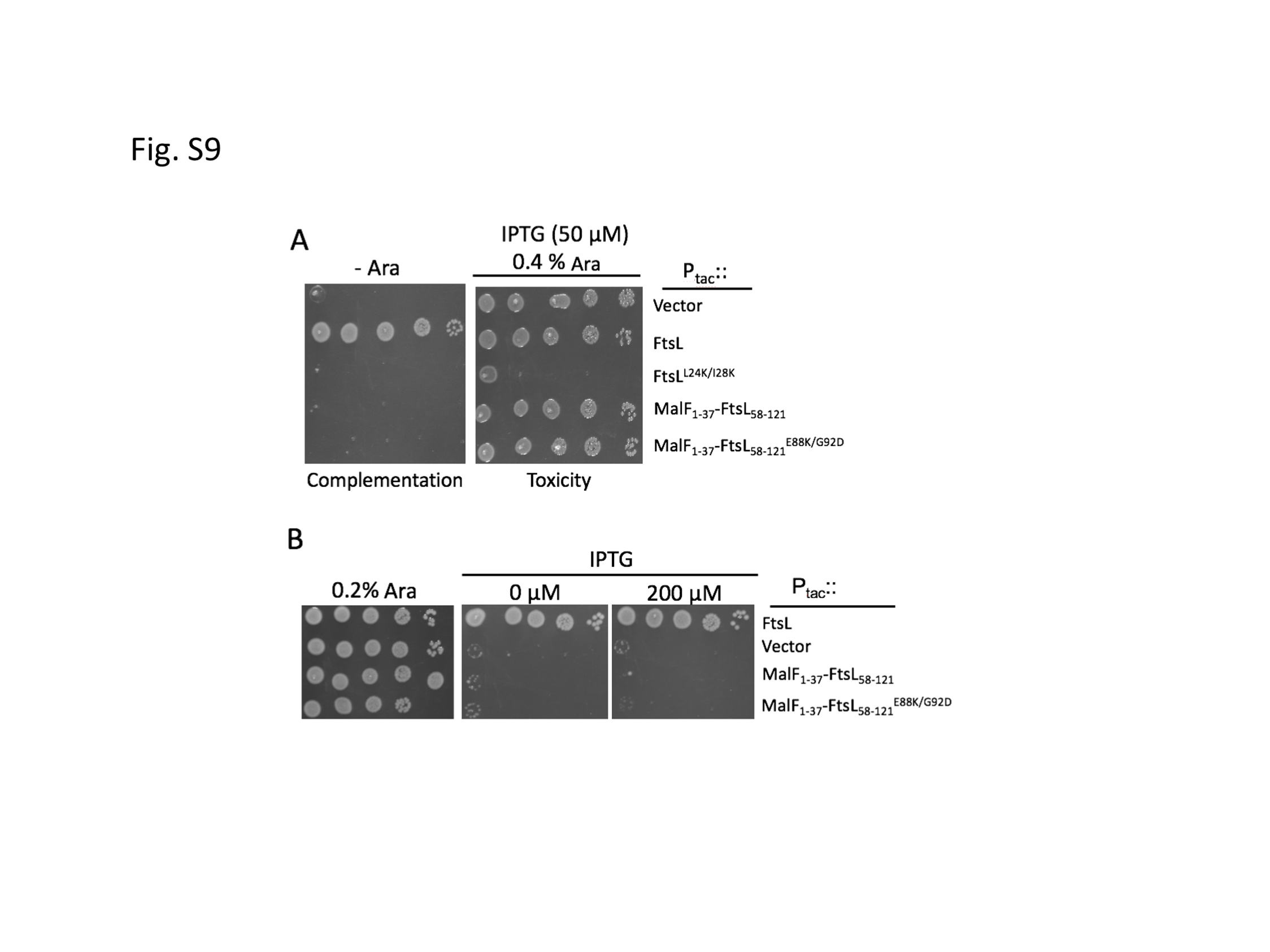

Supplement: FIG S9 [file mBio.03012-20-sf009.tif]
