# Supplementary material for: Essential Role for FtsL in Activation of Septal Peptidoglycan Synthesis
Source: mBio. 2020 Dec 8;11(6):e03012-20. doi: 10.1128/mBio.03012-20 (PMC7733951; doi:10.1128/mBio.03012-20)
Supplement: TABLE S1 [file mBio.03012-20-st001.docx]

Table S1. Strains and plasmids used in this study.

A. Strains

| Strains | Genotype | Source/ reference |
| --- | --- | --- |
| AM1992 | \|  \| *ΔrecA1921::aadA* \| \| --- \| --- \| | E. coli Stock center |
| BL155/pBL194 | TB28, ftsB<>aph / pSC101^TS^, aadA repA^TS^ P_syn135_::*gfp-ftsB* | (1) |
| BL156/pJH2 | TB28, *ftsL::kan*/pJH2 (P_syn135_::*ftsL*) | (1) |
| BL167 | TB28, *ftsB^E56A^* | (1) |
| CH34/pMG20 | *ftsN::kan* | (1) |
| DHM1 | BATCH reporter strain  MG1655 *F-, cya-854, recA1, endA1, gyrA96 (Nal^r^), thi1, hsdR17, spoT1, rfbD1, glnV44(AS)* | (2) |
| EC912 | W3110 ΔlacU169 gal-490 ftsW::kan/pDSW406 | (13) |
| EC436 | *MC4100* *Δ* (λ*attL‐lom*)::*bla lacI*^q^ *P*_207_‐*gfp‐ftsI* | (3) |
| JS238 | MC1061 *malPp*::*lacI*^Q^ *srlC*::Tn10 *recA1* | (4) |
| KTP1 | W3110 *recA::aadA* | P1 on AM1992 X W3110, select Spc^R^ |
| MCI23 | MC4100 *ftsI23* (Ts) | (5) |
| MCI23 recA | MC4100 *ftsI23* (Ts) *recA::aadA* | P1 on AM1992 X MCI23, select Spc^R^ |
| PK168-1 | BL167 *ftsL::kan recA::aadA/*pSD296 | P1 on BL156/pJH2 X BL167/pSD296 select Kan^R^; P1 on AM1992 select spc^R^ |
| PK247-4 | SD247 *ftsL::kan/*pSD296 | P1 on SD399 X SD247, select kan^R^ |
| PK4-1 | *W3110 ftsL::kan/pKTP108* | P1 on BL156 (pJH2) X W3110/pKTP108 |
| S3 | W3110 *leu*::Tn*10* | (6) |
| SD247 | W3110 *ftsW^M269I^* | (7) |
| SD247-1 | W3110 *recA::aadA ftsW^M269I^* | P1 on KTP1 X SD247 select Spc^R^ |
| SD285 | W3110 *leu::*Tn*10 bla lacI*^q^ *P*_207_‐*gfp‐ftsI* | P1 on EC436 X S3 (select Amp^R^) |
| SD399 | *W3110, ftsL::kan/*pSD256 | (7) |
| SD439 | W3110*, ftsL::kan*/pSD296 | (7) |
| SD488 | S3 *ftsW^E289G^* | Integration and resolution of pSD257 in S3, screen small cell phenotype and Spc^S^ |
| W3110 | WT | Lab collection |

B. Plasmids

| Plasmid | Genotype | Origin | Source/reference |
| --- | --- | --- | --- |
| pBAD33 | *cat* P*_ara_* | p15A | (8) |
| pBL154 | *aadA* pSC101/*repA^TS^* P_syn135_::*ftsN* | pSC101 | (1) |
| pDSW406 | *cat* pBAD33 P*_ara_::ftsW* | P15A | (13) |
| pDSW208 | bla lacI^q^ P_204_*::gfp* | ColE1 | (3) |
| pDSW210 | bla lacI^q^ P_206_*::gfp* | ColE1 | (3) |
| pGB2 | *aadA vector* | pSC101 | (9) |
| pJF118EH | bla lacI^q^ P_tac_*::vector* | ColE1 | (10) |
| pKTP100 | bla pJF118EH P*_tac_::ftsL* | ColE1 | This study |
| pKTP100* | bla pJF118EH P*_tac_:: ftsL^L86F,E87K^* | ColE1 | This study |
| pKTP101 | bla pJF118EH P*_tac_::ftsB* | ColE1 | This study |
| pKTP103 | bla pJF118EH P*_tac_::malF^1-37^ ftsL^58-121^-6xhis* | ColE1 | This study |
| pKTP104 | bla pQE80L P_T5_::*ftsL* | ColE1 | This study |
| pKTP105 | bla pQE80L P_T5_::*ftsL^30-121^* | ColE1 | This study |
| pKTP106 | *cat* pBAD3*3* P_ara_*::* *ftsL* | p15A | This study |
| pKTP107 | *cat* pBAD3*3* P_ara_*::* *ftsL^30-121^* | p15A | This study |
| pKTP107X | *cat* pBAD3*3* P_ara_::*ftsL^Δ^*^1-30^-*6Xhis* | P15A | This study |
| pKTP108 | *aadA* pBL154 repA^TS^ P_syn135_*::* *ftsL* | pSC101 | This study; ftsN in pBL154 replaced with *ftsL* |
| pKTP109 | *cat* pBAD3*3* P_ara_*::* *ftsI* | P15A | This study |
| pKTP110 | pDSW406 [P_ara_::*ftsW*] | ColE1 | This study |
| pMG20 | *cat* pBAD33 P_ara_::^SS^torA-bfp *ftsN^71-105^* | P15A | (1) |
| pND16 | *aadA* P*_ftsK_::* *ftsW-ftsK^179-1329^* | pSC101 | (11) |
| pND16* | *aadA* P*_ftsK_::* *ftsW^M269I^-ftsK^179-1329^* | pSC101 | This study |
| pQE80L | bla *lacI* P_T5_ | ColE1 | Qiagen |
| pSD256 | *aadA repA^ts^* P_ftsw_:*:ftsL* | pSC101 | (7) |
| pSD257 | *aadA repA^ts^* P*_ftsW_::ftsW* | pSC101 | This study |
| pSD257* | *aadA repA^ts^* P*_ftsW_::ftsW^M269I^* | pSC101 | This study |
| pSD296 | *cat* pBAD33 P_ara_*::ftsL* | p15A | This study |
| pSD296-2 | *cat* pBAD33 P_ara_*::ftsL^L86F,E87K^* | p15A | This study |
| pSD296-3 | *cat* pBAD33 P_ara_*::ftsL^L24K/I28K^* | p15A | This study |
| pSEB417 | bla pDSW208 P_204_*:*:*ftsN* | ColE1 | (12) |
| pSEB420 | bla pDSW208 P_204_*:*:*ftsI* | ColE1 | (12) |
| pSEB422 | bla pDSW208 P_204_*:*:*ftsL* | ColE1 | (12) |
| pSEB429 | bla pDSW208 P_204_:: *ftsW* | ColE1 | (12) |
| pSEB429-I | bla pDSW208 P_204_:: *ftsW^M269I^* | ColE1 | This study |
| pSEB453 | bla P*_ftsN_::* *malG^1–33^–ftsN^46–319^* | ColE1 | This study |
| pUT18C | *bla* BACTH vector T18 ORF | ColE1 | (2) |
| pKT25 | *aph* BACTH vector T25 ORF | p15A | (2) |
| pUT18C-zip* and pKT25-zip* | BACTH control plasmids |  | (2) |
| pKT25-ftsW | *aph* P_lac_*::cya^T25^-ftsW* | p15A | This work |
| pKT25-ftsW* | *aph* P_lac_*::cya^T25^-ftsW^M269I^* | p15A | This work |
| pKT25-ftsI | *aph* P_lac_*::cya^T25^-ftsI* | P15A | This work |
| pKT25-ftsQ | *aph* P_lac_*:: cya^T25^-ftsQ* | P15A | This work |
| pUT18C-ftsL | *bla* P_lac_::*cya^T18^*-*ftsL* | ColE1 | This work |
| pUT18C-ftsL^Δ1-30^ | *bla* P_lac_::*cya^T18^*-*ftsL*^Δ1-30^ | ColE1 | This work |

*Leucine zipper

References

1. Liu B, Persons L, Lee L, & de Boer PA (2015) Roles for both FtsA and the FtsBLQ subcomplex in FtsN-stimulated cell constriction in Escherichia coli. *Mol Microbiol* 95(6):945-970.

2. Karimova G, Dautin N, & Ladant D (2005) Interaction network among Escherichia coli membrane proteins involved in cell division as revealed by bacterial two-hybrid analysis. *J Bacteriol* 187(7):2233-2243.

3. Weiss DS, Chen JC, Ghigo JM, Boyd D, & Beckwith J (1999) Localization of FtsI (PBP3) to the septal ring requires its membrane anchor, the Z ring, FtsA, FtsQ, and FtsL. *J Bacteriol* 181(2):508-520.

4. Pichoff S & Lutkenhaus J (2007) Identification of a region of FtsA required for interaction with FtsZ. *Mol Microbiol* 64(4):1129-1138.

5. Dai K, Xu Y, & Lutkenhaus J (1993) Cloning and characterization of ftsN, an essential cell division gene in Escherichia coli isolated as a multicopy suppressor of ftsA12(Ts). *J Bacteriol* 175(12):3790-3797.

6. Shen B & Lutkenhaus J (2009) The conserved C-terminal tail of FtsZ is required for the septal localization and division inhibitory activity of MinC(C)/MinD. *Mol Microbiol* 72(2):410-424.

7. Du S, Pichoff S, & Lutkenhaus J (2016) FtsEX acts on FtsA to regulate divisome assembly and activity. *Proc Natl Acad Sci U S A* 113(34):E5052-5061.

8. Guzman LM, Belin D, Carson MJ, & Beckwith J (1995) Tight regulation, modulation, and high-level expression by vectors containing the arabinose PBAD promoter. *J Bacteriol* 177(14):4121-4130.

9. Churchward G, Belin D, & Nagamine Y (1984) A pSC101-derived plasmid which shows no sequence homology to other commonly used cloning vectors. *Gene* 31(1-3):165-171.

10. Furste JP*, et al.* (1986) Molecular cloning of the plasmid RP4 primase region in a multi-host-range tacP expression vector. *Gene* 48(1):119-131.

11. Dubarry N, Possoz C, & Barre FX (2010) Multiple regions along the Escherichia coli FtsK protein are implicated in cell division. *Mol Microbiol* 78(5):1088-1100.

12. Pichoff S, Du S, & Lutkenhaus J (2015) The bypass of ZipA by overexpression of FtsN requires a previously unknown conserved FtsN motif essential for FtsA-FtsN interaction supporting a model in which FtsA monomers recruit late cell division proteins to the Z ring. *Mol Microbiol* 95(6):971-987.

13. Mercer, KL & Weiss, DS (2002) The Escherichia coli cell division protein FtsW is required to recruit its cognate transpeptidase, FtsI (PBP3), to the division site. *J Bacteriol* 184(4):904-912.
